# Key Stakeholders’ Experiences and Perceptions of Virtual Reality for Older Adults Living With Dementia: Systematic Review and Thematic Synthesis

**DOI:** 10.2196/37228

**Published:** 2022-12-23

**Authors:** Aisling Flynn, David Healy, Marguerite Barry, Attracta Brennan, Sam Redfern, Catherine Houghton, Dympna Casey

**Affiliations:** 1 School of Nursing and Midwifery Aras Moyola Galway Ireland; 2 School of Psychology University of Galway Galway Ireland; 3 School of Information and Communication Studies, ADAPT Centre University College Dublin Dublin Ireland; 4 Engineering and Informatics University of Galway Galway Ireland

**Keywords:** virtual reality, VR, dementia, experience, perception, qualitative evidence synthesis (QES), thematic synthesis

## Abstract

**Background:**

Technology is increasingly being used and evolving in the dementia care landscape. One such technology that has gained traction over the last decade is virtual reality (VR). VR is being applied in many areas of dementia care, including cognitive assessment and training, reminiscence therapy, music therapy, and other recreational VR applications. Despite the plethora of applications, they are often not shaped by the experiences and perceptions of older adults living with dementia. Currently, there is no qualitative evidence synthesis (QES) to explore this area. This review aimed to provide qualitative evidence supporting existing systematic reviews in this area.

**Objective:**

The aim of this QES was to explore key stakeholders’ experiences and perceptions of VR for older adults living with dementia. It aimed to explore the barriers and facilitators to VR use and provide recommendations for future design and implementation.

**Methods:**

QES was used, which involved a systematic search of 6 databases to identify studies that qualitatively explored key stakeholders’ experiences and perceptions of VR for older adults living with dementia. Thematic synthesis was used to integrate the findings of 14 studies (from 15 reports). The Critical Appraisal Skills Programme tool was used to assess the methodological quality of the included studies. The confidence placed in the review findings was assessed using the GRADE-CERQUAL (Confidence in the Evidence from Reviews of Qualitative research).

**Results:**

A total of 15 reports from 14 studies were included in the review, consisting of a range of levels of VR immersion, stages of dementia, and care contexts. Three analytical themes were generated: stepping into virtuality, a virtual world, and returning to reality. The results indicate the importance of sensitively designing and introducing VR to this population, as older adults living with dementia often have no prior experience of using this technology. VR can be a positive experience for older adults living with dementia and can provide meaningful interactions, positive expressions, and long-term impacts on everyday functioning. However, it should be acknowledged that some negative associations must be accounted for before, during, and after use.

**Conclusions:**

This review highlights the positive implications as well as negative associations of VR use. It emphasizes the need for VR design and implementation driven by the needs and views of older adults living with dementia as well as with other key stakeholders. Future research needs to explore the vital role that older adults living with dementia can play in the design process and how they can be empowered to meaningfully design and use this technology.

## Introduction

### Background

Dementia is a progressive disease characterized by the deterioration in memory, executive functioning, behavior, and everyday functioning [1]. Globally, the number of people living with dementia is rapidly increasing with an estimated 50 million people living with the condition at present [2,3]. This figure is expected to rise to 139 million in 2050. There is currently no cure for dementia or a treatment that can change the progressive nature of the disease [4,5]. A traditional medical model of practice has emphasized the role of pharmacological interventions to address the symptoms associated with dementia [6-8]. However, a shift to a more biopsychosocial model of dementia advocates for the use of nonpharmacological interventions using person-centered, wellness and enablement approaches to dementia care [8-10]. The outcomes of such interventions have been categorized by the National Institute for Health and Care Excellence as those that reduce responsive behaviors; those that maintain or improve one’s functional capacity; and those that aim to reduce comorbid emotional disorders [9,11]. There is a strong evidence base to support the use of interventions such as cognitive and sensory stimulation, exercise, music therapy, reminiscence, and validation therapy [9].

Considering that there is currently no cure for dementia, strategies to support the psychosocial needs of older adults living with dementia are important. Current and emerging technologies aim to support people living with dementia throughout their disease trajectory [4,12,13]. Digital technology has been cited as a means of delivering nonpharmacological approaches to address the noncognitive aspects of dementia [14-16]. Technology-mediated nonpharmacological therapies include reminiscence and music therapy [15,17,18]. Such technologies are moving away from traditional, widely researched, passive applications, such as ambient-assisted living and monitoring systems, to more active technologies. Active applications can promote meaningful activities and include social robots, tablets, PCs, and virtual reality (VR) systems [17,19]. These more active technologies are relatively novel and underresearched in the dementia care landscape [17] but indicate promising benefits for promoting the well-being of people living with dementia [16,20]. This review focuses on VR as one such active technology.

VR is a means of relocating people to virtual places where they can participate in events and activities [21]. VR exists on a spectrum and can vary from nonimmersive VR to fully immersive systems. Nonimmersive systems often act as a “window” to the virtual world by interacting through a flat-screen PC [22,23]. Semi-immersive systems have an extended field of view that accommodates additional sensory modalities for interaction in a virtual environment (VE). Fully immersive systems are referred to as those in which the user is present in a VE using a head-mounted display (HMD) or a Cave Automatic Virtual Environment [22,23]. The hardware and software specifications can vary across levels of immersion with fully immersive systems occluding the physical world.

The distinguishing factor between VR across the spectrum of immersion and other technologies is the level of participation, where the user has the opportunity to participate in the VE rather than merely observe it [21]. VR systems operate on a spectrum of interactivity that varies depending on the aims of the user, scope of the system, and equipment used. VR experiences can be classified as passive, exploratory, and interactive [24]. Passive interaction refers to low interaction, where users have the freedom of where to look, for example, 360º video [22]. Exploratory interaction allows the user to move around the VE and the user has freedom of where to look. However, this does not accommodate touch [22]. The interactive level enables the user to “explore, control and modify” the VE [22]. This stimulates several senses and accommodates haptic feedback and interaction [22]. This review explores VR use across the spectrum of immersion and interaction, specifically in the context of people living with dementia aged >60 years.

### Why Is It Important to Do This Review?

VR shows promise in its potential to provide positive experiences for older adults living with dementia. Research illustrates the impact of VR on sociability, quality of life, activities of daily living, cognitive health, and independence [25-28]. Despite the positive potential of VR, there are also negative side effects associated with its use such as simulator sickness and disorientation [29]. Although there has been a significant increase in VR use over the past decade, there is a lack of high-quality, systematic research. Although reviews have been undertaken investigating the effectiveness, feasibility, acceptability, and usability aspects of VR for older adults and people living with dementia, such reviews have mainly explored quantitative or mixed method reviews, the latter of which have typically lacked a systematic approach. No qualitative synthesis has explored the experiences and perceptions of key stakeholders such as people living with dementia, family members, health and social care professionals, and other facilitators of VR. Therefore, a systematic review and thematic synthesis is required. Qualitative evidence synthesis (QES) affords insights into the experiences and perceptions of VR from a multistakeholder perspective, thus informing future design and implementation of VR for this population [30].

Systematic reviews that use a qualitative synthesis approach are in keeping with an accelerated move beyond reviews that focus on the effectiveness to reviews that synthesize evidence related to experiential elements of certain phenomena [31,32]. The voice of people living with dementia and their caregivers are increasingly being prioritized both in research and policy to ensure results are driven by their experiences and needs [33-37]. QES can capture stakeholders’ multiple perspectives across a range of studies, which may be lost when a study is explored in isolation or through a quantitative synthesis approach [34,35,38]. For the remainder of this paper, the term QES is used as an encompassing term for a systematic review and thematic synthesis.

### How This Review Might Inform or Supplement What Is Already Known in This Area

There are several narrative and systematic reviews that provide a useful reference point for this QES, and such reviews have taken a quantitative and mixed method approach to the exploration of VR [25-28]. In such cases, VR has not focused on the full spectrum of immersion; rather, it has mainly focused on semi-immersive and fully immersive systems. This makes it difficult to draw comparisons between the various modes of delivery (ie, projectors vs HMDs). Previous reviews also lack an experiential focus, and this review aimed to explore this area qualitatively. As this is an emerging and fast-paced area, regular updated reviews are imperative to keep up to date and relevant. Reviews also vary in their exploration of the technology, with no specific focus on older adults living with dementia nor on the perspectives of other stakeholders or the spectrum of VR. Age-related factors regarding VR use vary between people living with early onset dementia and older adults with dementia. This review focuses on the experience and perceptions of VR use in the latter cohort through a multistakeholder lens [39-42] and provides design recommendations that align with the review objectives and findings of QES to supplement this area of research.

### Objectives

The following are the objectives of this QES:

- To explore key stakeholders’ experiences and perceptions of using VR technology for older adults living with dementia.
- To identify perceived facilitators and barriers to the use of VR technology for older adults living with dementia.
- To develop recommendations for the development of future VR experiences for older adults living with dementia.

## Methods

### Overview

The Effective Practice and Organization of Care protocol and review template for QES was used to guide the review process in this study [43]. This review adhered to the Enhancing Transparency of Reporting the Synthesis of Qualitative Research framework [44]. The QES protocol was registered in the International Prospective Register of Systematic Reviews (CRD42020208228) and published in a peer-reviewed journal [45].

The SPICE framework (setting, population, intervention, comparison, and evaluation) was used to determine the review question and search terms used to answer the review aims [46]. Systematic reviews were scoped to determine previously used dementia-specific and VR-related terms relevant to the review [47,48].

### Criteria for Considering Studies for This Review

#### Types of Studies

Primary studies that used qualitative research design were included. Studies that used qualitative methods of data collection such as interviews, observations, and focus groups were eligible for inclusion. Studies that did not report primary research such as other systematic reviews were excluded. Mixed methods studies that used a qualitative method of data collection and in which qualitative data could be extracted were also eligible for inclusion. This review included those eligible studies that had been published through to October 2020. There was no starting time limit for the inclusion of studies.

#### Topic of Interest

The studies included key stakeholders’ experiences and perceptions of VR technology use for older adults living with dementia. Data related to the qualitative discussion of the views, perceptions, or experiences of stakeholders regarding the use of VR in the results or discussion section of the studies were considered eligible. Key stakeholders related to older adults living with dementia, family members, health and social care professionals, and other facilitators of VR. VR technology for inclusion was classified as nonimmersive, semi-immersive, or fully immersive [23]. Studies from any setting such as Residential Aged Care Facilities (RACFs) and community or acute settings were eligible for inclusion.

### Medical Subject Headings (MeSH)

Searches were designed in consultation with an information specialist at University of Galway. A scoping search was completed to identify suitable keywords, MeSH, and suitable literature (Multimedia Appendix 1).

#### Electronic Searches

A systematic search was conducted using the Scopus, Compendex, AgeLine, CINAHL, MEDLINE, and PsycINFO databases in October 2020 (Multimedia Appendix 1). Variations of search terms and MeSH terms were used according to database conventions. No year limit was applied to the search strategy.

#### Searching Other Resources

Additional search methods were used to avoid omitting unindexed articles [49]. Google Scholar and ProQuest were searched and the first 200 articles were screened for eligibility. Handsearching of reference lists was also performed to locate relevant articles through citation chaining. Google Scholar’s “cited-by” function was used to perform a forward citation search. All records were downloaded to Endnote ×9 (Clarivate), and duplicate records were removed.

### Selection of Studies

**Figure 1:**
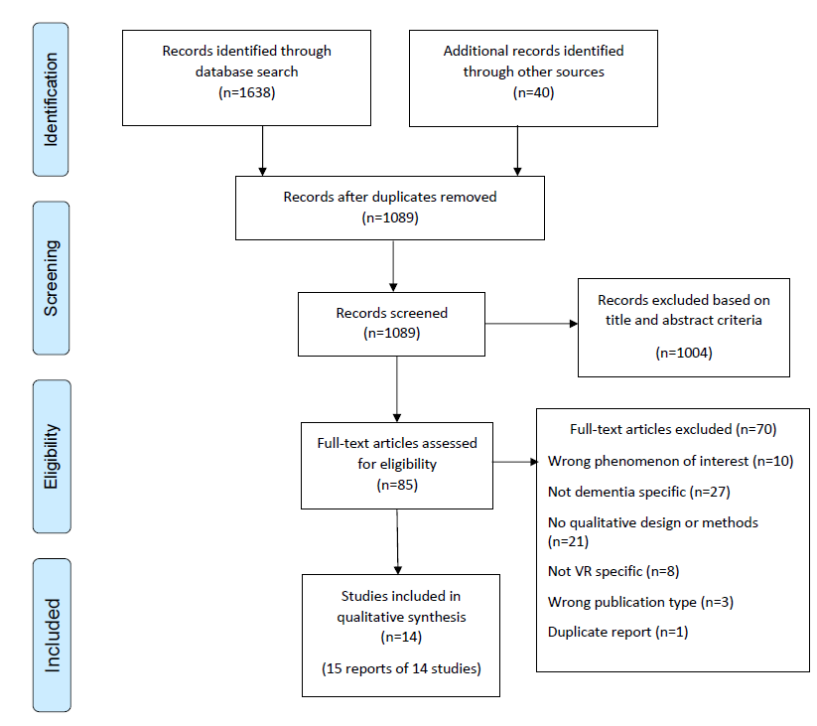
PRISMA (Preferred Reporting Items for Systematic Reviews and Meta-Analyses) flow diagram. VR: virtual reality.

A total of 2 authors (AF and DH) independently completed 100% of title and abstract and full-text screening using the Rayyan screening software. Details of the screening process can be found in Multimedia Appendix 2. Inclusion and exclusion criteria were used to guide the screening process (Multimedia Appendix 3). Screening was completed with blinding turned on. Disagreements were resolved through consultation with a third reviewer (CH). A PRISMA (Preferred Reporting Item for Systematic Reviews and Meta-Analyses) flow diagram (Figure 1) illustrates the search results, screening process, included studies, and excluded studies with a rationale for exclusion.

### Language Translation

The titles and abstracts that were not in the research team’s native language were initially translated using Google Translate. This study did not yield any papers that required full-text translation.

### Data Extraction

Two reviewers (AF and DH) completed data extraction using a bespoke form devised for this review. The form was piloted and refined by AF and DH. Data extracted for synthesis was in accordance with the approaches of Thomas and Harden [50] and Noyes et al [51], acknowledging the diversity in defining what constitutes “data” for primary qualitative studies.

For this review, data were considered as direct participant quotations and observations. Indirect findings from the authors such as observations and themes, were also extracted. Data related to the review aims were extracted. Data were not extracted in cases where studies had a broader focus or included data from adults aged <60 years without a diagnosis of dementia. Data extraction for each report can be found in Textbox 1.

All extracted data were entered into NVivo (version 12; QSR International) [52]. Data extraction was compared, and conflicts were resolved in consultation with 2 authors (DC and CH), where necessary. Details of the characteristics of the included studies can be found in Multimedia Appendix 4.

**Data extraction for each report.**

**The following data were extracted for each report:**

- Author
- Year
- Country
- Study aim
- Study design
- Key stakeholders
- Sample size
- Type of dementia
- Physical abilities of older adult with dementia
- Previous virtual reality (VR) experience
- VR use context
- Level of immersion of VR system
- Hardware specifications
- Software specifications
- Procedural aspects
- Results or findings

### Assessment of Methodological Limitations of Included Studies

The methodological limitations of the included studies were assessed by 2 authors (AF and DH) using the Critical Appraisal Skills Programme Qualitative Checklist [53] (Multimedia Appendix 5). This was completed in parallel with data extraction. Disagreements were resolved through consultation with either DC or CH. Case classifications in NVivo were used to detail the study characteristics and Critical Appraisal Skills Programme responses [52].

### Data Management, Analysis, and Synthesis

The RETREAT (Review question-Epistemology-Time or Timescale-Resources-Expertise-Audience and purpose-Type of Data) framework guided the authors’ decision to apply thematic synthesis to the QES [54]. The framework identifies the appropriate method for QES based on the following 7 tenets: review question, epistemology, time or timescale, resources, expertise, audience, and purpose-type of data [54]. On the basis of the given criteria, thematic synthesis as per Thomas and Harden [50] was chosen for this review for several reasons: its focus on answering multiple review questions, its broad focus on a wider audience, its accommodation for both thick and thin data, and its applicability to the researcher’s experience with QES.

The author followed the 3 core stages of thematic synthesis: line-by-line coding, the development of descriptive themes, and the establishment of analytical themes [50]. Line-by-line coding results in “free codes,” which are then combined into descriptive themes closely related to the primary data. The analytic codes move past this descriptive element and aim to “go deeper” to address the review aims and develop a deeper level of interpretation [50,55].

The stages of thematic synthesis were managed in NVivo, resulting in an audit trail to enhance transparency [52]. One reviewer (AF) inductively coded 15 reports line by line. These codes were then organized into descriptive themes. Two reviewers (AF and CH) met to discuss the descriptive themes and agreed on how best to approach the development of the analytical themes. Focusing on the aims of the review, 1 reviewer (AF) generated analytical themes. Analytical themes were discussed and iteratively refined based on consultation with review team members (DH, DC, and CH). Furthermore, 2 people living with dementia, as part of a public and patient involvement advisory team, were given an opportunity to review the themes and provide feedback. This feedback informed the iterations of the analysis and narrative findings.

### Assessing Confidence in the Review Findings

Two review authors (AF and DH) applied the GRADE-CERQual (confidence in the evidence from reviews of qualitative research) tool to assess confidence in each of the key findings [56]. GRADE-CERQual assesses confidence in evidence based on the following 4 key components: methodological limitations, coherence, adequacy, and relevance [56]. Confidence was assessed as high, moderate, low, or very low. The final assessment was based on a consensus among the review authors and in consultation with CH (Multimedia Appendices 6 and 7).

### Summary of Qualitative Findings Table and Evidence Profile

A summary of the findings and the assessment of confidence is presented in Multimedia Appendix 6 and a full evidence profile is presented in Multimedia Appendix 7.

### Review Author Reflexivity

The research team came from diverse backgrounds, including occupational therapy (OT; AF), human-computer interaction (HCI) research (AF), health psychology (DH), and nursing (DC and CH). The research team members have a range of expertise and experience in the use of qualitative methods. Each member, except for one (DH), had experience working with people living with dementia in both research and clinical settings. CH and DC are experienced in the application of QES and thematic synthesis, whereas CH has extensive expertise in the use of GRADE-CERQual. CH and DC have published several QES papers focused on the area of dementia care and, therefore, have had unique insights and contributions on the complexities and pragmatic aspects of completing such reviews. Two members are conducting empirical research related to VR design for older adults (DH) and people living with dementia (AF). Two team members research VR development (SR and AB).

During screening, the team was in contact to resolve conflicts as they arose. Two members (AF and DH) completed screening, data extraction, and assessment of methodological limitations independently and were in regular contact with one another, discussing how their backgrounds in health psychology and OT influenced their decisions. An open communication process eliminated the potential bias that some members may have had when making decisions for the inclusion and exclusion of papers or deriving themes based on previous clinical and research experience.

## Results

### Results of the Search

A total of 15 reports of 14 studies satisfied the inclusion criteria.

### Description of the Studies

These studies were conducted between 2018 and 2020. All studies used a qualitative or mixed method approach to the study design and data collection methods. However, there was variance in the reporting of the specific qualitative methodology used. Qualitative data from 6 mixed method studies were extracted. Studies were completed in Australia [57-59], the United Kingdom [60-64], Canada [65], the Netherlands [66], the United States [67], France [68], Germany [69], Cyprus [70], and South Korea [71]. Study settings included RACF [57-59,63,64,66,71], acute inpatient settings [61,62,70], community day-care settings [60,65,68,69], and hospice settings [67]. A total of 234 stakeholders were reported in the included studies, which consisted of 199 older adults living with dementia, 85 formal and informal caregivers (nursing, physiotherapy, OT, activity managers, and managerial staff), and 14 family members. It is important to note that the number of participants has not been reported in some studies. Therefore, the exact number of health and social care professionals and family members reported in this review was not representative. Such omissions were considered in relation to the overall contribution to the review findings during the GRADE-CERQual assessment [72]. Family members were also referred to as caregivers in this context.

The review included 7 fully immersive studies using HMDs and Google Cardboard [57,60-62,67,70,71]. Overall, 5 reports from 4 studies included semi-immersive systems using projectors and Microsoft Kinect [58,59,63-65]. Three studies included nonimmersive systems consisting of virtual and interactive environments displayed on a PC or television screen with interactivity supported through a mousepad and other controllers [66,68,69]. Experiences and perceptions of VR entail an array of concepts relating to the systems, including usability, acceptance, acceptability, adoption, and feasibility. Details of the characteristics of the included studies can be found in Multimedia Appendix 4.

### Methodological Limitations of the Studies

A total of 15 reports were included: 8 were assessed as having no or very minor concerns [57-62,67,69], 4 studies were assessed as having moderate concerns [63,65,66,70], and 3 studies were assessed as having serious concerns [64,68,71]. Many of the included studies failed to mention important procedures and demographic information related to VR use such as informed consent procedures or the stage of dementia. A lack of reporting on the relationship between the researcher and the participants was also observed in most of the included studies. It is difficult to ascertain whether this nonreporting is a consequence of not completing certain procedures. People living with dementia are considered a vulnerable population [73,74]; thus, researchers and facilitators of VR need to work in an ethical manner to ensure transparency in the reporting of ethical procedures. The detailed rationale for the authors’ decisions is presented in Multimedia Appendix 5.

### Confidence in the Review Findings

GRADE-CERQual was used to assess the qualitative findings (Multimedia Appendices 6 and 7). Six findings were assessed as high confidence and 10 as moderate confidence. The findings with a moderate confidence rating exhibited concerns related to the methodological limitations and relevance of the supporting studies. Methodological concerns are related to the lack of reporting on research design, ethical procedures, and researcher reflexivity. Regarding relevance, there were disproportionate and unclear samples of older adults living with dementia, formal and informal caregivers, and other stakeholders. Despite the stakeholders providing the information, the phenomenon of interest was always related to VR use for older adults living with dementia, and there were sufficient perspectives and experiences to ensure minimal concerns regarding the adequacy and confidence in the findings. The rationale for the GRADE-CERQual assessment is provided in a summarized and full evidence profile (see Multimedia Appendices 6 and 7).

### Review Findings

**Figure 2:**
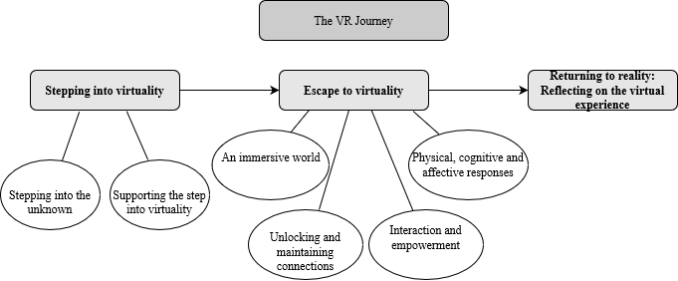
Flowchart of derived analytic and descriptive themes depicting the VR journey. VR: virtual reality.

Three analytical themes were established to describe key stakeholders’ experiences and perceptions of VR for older adults living with dementia. Analytical themes were further explained through several subthemes generated from descriptive themes. These findings represent a range of stakeholder views in several contexts and at different stages of dementia diagnosis. A diagram illustrating a summary of the derived themes is presented in Figure 2 and provides additional context to the findings. Table 1 and Multimedia Appendix 8 present supporting primary data from the original papers.

**Table 1:** Supporting quotations for the analytical themes and their subthemes.

Analytical theme and subtheme			Quotation
**Stepping into virtuality**			
	Stepping into the unknown	“Caregivers were unsure whether people with dementia would try HMD^a^-VR^b^ at all.” [61]	
	Supporting the step into virtuality	“With encouragement from her daughter it seemed a lot easier to ask Lucy to try it [HMD] on” [60]; “I think by having a nurse and a physiotherapist come in, it gives a bit of reassurance for the resident” [58]	
**Escape to virtuality**			
	An immersive world	“VWs^c^ [...]triggered the overall feeling of ‘being in a garden’.” [63]; “Most participants reported a high level of presence during the interviews, reporting that it felt ‘real’ or ‘like they were in there’.” [62]	
	Unlocking and maintaining connections	“VWs [virtual worlds] allow the residents to temporarily step outside of their closed physical environment of long-term care facilities and transport them to a (albeit virtual) world of reminiscence” [63]; “The interaction...because we have got two levels, those downstairs never meet people from upstairs...and they got to know each other” [58]	
	Interaction and empowerment	“the installation gives them a feeling of being in the control and meaningfulness.” [66]; “Mum was using her hands to control the movement. It means she’s got control of something in her life, that control element. What other control has she really got?” [FF6] [59]	
	Physical, cognitive, affective	“The majority of the residents seem to really enjoy it. I see their faces smiling, and they seem quite relaxed with it” [SF1] [59]; Post VR Observations [...] Commented, “It was the best day ever” [...] “Best day I’ve ever had.” [PWD3, Observations, 1] [62]	
**Returning to reality: Reflecting on the virtual experience**			
		“It’s a one-time experience, you don’t need it twice” [67]; When asked if she would continue to use the system after the study ended, she replied “yes, it is a good memory training and it gives it a structure to everyday life.” [69]	

^a^HMD: head-mounted display.

^b^VR: virtual reality.

^c^VW: virtual world.

### Stepping Into Virtuality

This theme relates to stakeholders’ anxieties and concerns regarding anticipation of VR use. All studies reported that older adults living with dementia had no prior experience of using VR, consolidating the need to carefully introduce VR to this population and other stakeholders such as informal and formal caregivers. The subthemes are stepping into the unknown and supporting the step into virtuality.

#### Stepping Into the Unknown

This subtheme explored the concerns, worries, and anxieties experienced by older adults living with dementia and formal and informal caregivers in anticipation of VR use. Formal caregivers and family members across 4 studies reported skepticism and concerns regarding the stage of dementia, physical functioning, and its impact on enjoyment [59]; possible negative emotional impact [61]; VR being perceived as lonely or scary [61]; or preconceptions surrounding the potential disorientation, perseveration, confusion, or risk of falling while using VR. Caregivers questioned why residents could not visit physical locations rather than virtual ones. They also expressed that visiting virtual locations in VR may prompt residents to request visits to inaccessible physical locations after use. Older adults living with dementia also have concerns regarding the appearance of VR systems, leading to a refusal to try VR [70]. Those who agreed to trial the fully immersive HMD cited it as being large, intimidating, scary, and unnatural, and declined further use to avoid looking foolish and to protect their dignity [60].

To appropriately introduce VR to older adults living with dementia and their informal and formal caregivers, there is a need for adequate setup and implementation procedures to ensure eligibility, reassurance, safe setup, and use. Eligibility and safety screening were advocated by facilitators in studies to identify those most suited to VR use and accommodate their needs [58,62]. Care staff and family members gradually introduced VR to older adults living with dementia by using VR in their own bedrooms before use in a common area, showing videos and images of the system, and having time to try the headsets before sustained use [58,60]. Facilitators of VR, including researchers and care staff, noted the value of easy-to-set-up, portable hardware systems with troubleshooting guides [62]. In particular, 3 care staff members in a study reported difficulty setting up body tracking systems for those seated or using wheelchairs [59]. Two studies ensured safe reaching and rotation by positioning the equipment in the center of the tracking space [57,62].

#### Supporting the Step Into Virtuality

Supporting the step into virtuality subtheme explored the support and encouragement of informal and formal caregivers in providing a gateway to VR for older adults living with dementia. It explored the role of facilitators before and during VR use. Support and encouragement were manifested through verbal feedback, prompts, physical assistance, and readjustment before and during the system use. Facilitators varied across studies and included researchers, physiotherapists, nursing staff, informal caregivers, and activity coordinators.

The trusted relationship between older adults living with dementia and their caregivers served as a means of encouraging VR use [60,69]. Three studies referred to persons living with dementia requesting a family member’s presence when using VR [59,69,71]. The facilitator was perceived as a vital agent in monitoring engagement, encouraging interactivity, empowering, and reassuring older adults living with dementia when using VR [58,59,71]. Through the spectrum of immersion, the facilitator adapted VR in response to observations and feedback from older adults with dementia to sustain engagement, attention, and motivation [59,62-64,70]. Verbal encouragement and feedback regarding the upcoming tasks and interactions to be completed facilitated encouragement [70]. The facilitator often asked probing questions during the activity to gauge tolerance and enjoyment and to facilitate conversations for sustained use [60,63,67,70]. Akin to monitoring safety and interaction, the facilitator played a role in monitoring how the headset and VR equipment was tolerated, often physically readjusting the headsets or repositioning the person during VR use [57,70].

### Escape to Virtuality

This theme explored the experiential elements of VR use. Subthemes include an immersive world; unlocking and maintaining connections; interaction and empowerment; and physical, cognitive, and affective responses to virtuality*.* Subthemes mainly focused on observational data from staff and verbal feedback from older adults living with dementia and formal and informal caregivers.

#### An Immersive World

The included studies described the observations and verbal descriptions that indicated experiencing a sense of presence, immersion, or embodiment in the VE. This was evidenced across the spectrum of immersion, including 3 fully immersive, 2 semi-immersive, and 1 nonimmersive studies. A total of 8 older adults living with dementia in 3 studies reported VR as realistic and alluded to a sense of presence through descriptions of the content within the VEs [61-63]. Older adults living with dementia demonstrated the ability to suspend their disbelief and allow themselves to attend VEs, thus providing a sense of presence [61,62,71]. Furthermore, 3 older adults reported thinking they were cycling or rowing a boat in semi-immersive VEs [58,63]. One study noted that people living with dementia reported feeling embodied within a virtual body, whereas studies without avatars found that people living with dementia did not notice the absence of a virtual body [62]. There were instances where this sense of presence resulted in confusion between virtual and physical or real environments by one older adult living with dementia [63]. After using the system, one resident living with dementia sought trees from the VE in the physical space, illustrating its potential to exacerbate confusion [63].

#### Unlocking and Maintaining Connections

The unlocking and maintaining connections subtheme refers to connections established through VR use. VR served as a medium of virtual mobility or teleportation to different locations, affording older adults living with dementia opportunities to rekindle connections to their inaccessible present, past, and other individuals. The focus on person-to-person and person-to-object connections within the VE served as a medium for consolidating these connections.

Older adults living with dementia, family members, and caregivers cited VR as an escape from reality and a means to visit areas beyond their residence in the “inaccessible present” due to resource constraints and physical or cognitive decline [60-62,71]. Familiar locations and objects in the VE provided an excursion opportunity and connected people living with dementia to their locality or objects, such as animals [58,66].

VR enabled older adults living with dementia to experience a world of reminiscence, allowing them to connect and embrace their past across all levels of immersion [60,62,63]. Two inpatient studies noted VR as an opportunity to reproduce past events that cannot be replicated in the physical world or through routine RACF activities [62,66]. Connecting to the past sparked conversations about one’s life narrative with care staff and informal caregivers by sharing old photographs, detailing their past occupations, and hobbies [64,66]. One caregiver also considered a nonimmersive system to evoke the nurturing nature of their mother [63,66]. Another study observed that a male resident with dementia recreated past experiences through movement by taking up a boxing stance in a semi-immersive environment [63].

A total of 7 studies reported that VR provided an opportunity to connect with others both within and outside their facility through engagement in mutual activities, discussion of the session, and anticipation for subsequent sessions [58,61,63,64,67,69]. Several people living with dementia used the VE content as an opportunity to talk about life experiences after VR sessions with formal and informal caregivers and other people living with dementia [60,62-64,66,69]. Two family members noted that VR provided a shared social activity in their locality, thus connecting with themselves, peers, and formal caregivers [60].

#### Interaction and Empowerment

Many people living with dementia interacted in VR through passive and simplistic means by observing the VE through limited interaction, naming, pointing, and discussing objects with the facilitator. Formal caregivers reported that older adults living with dementia had the ability to interact independently, provided an adequate rest period, and incorporated an appropriate level of interaction. However, 4 studies reported that older adults with dementia became confused or distracted during interactions [63,65,68,70]. The care staff in 2 studies also reported dynamic interactions across subsequent VR sessions [61,62].

When the capabilities of the person living with dementia and interactivity within the VE were in harmony, a sense of empowerment and control was experienced. This was attributed to the agency that older adults living with dementia had to freely choose their preferred environments, interactions, and movement in the VE [58,60,62,63,66,71]. Having control over the system can facilitate self-efficacy and “draw people out of themselves” to explore their unlocked capabilities [59,69], providing a new lens for caregivers to recalibrate their knowledge of the person living with dementia [61,62]. The staff reported that this agency over interactions in the VE may be the only means of control in their lives. However, when this harmonious balance was not achieved, interactions served to hinder one’s sense of control [59,63]. Difficulty with interaction and a lack of control were attributed to issues such as nonintuitive button placement of the controllers [57,70], reduced dexterity and grip of older adults with dementia, inappropriate body tracking for wheelchair or seated users [57], disassociation between the controllers in the physical space and how they contributed to the VE [58], and difficulty with head movements and the tolerability of the headset [62,67,70].

#### Physical, Cognitive, and Affective Responses to Virtuality

The physical, cognitive, and affective responses to virtuality are related to the emotions and sensory effects of using VR. These include the enlivening effect of VR, calming effect of VR, and dynamic emotions and sensations associated with VR use. Older adults living with dementia experienced positive emotions and sensations during and after VR use.

Positive effects were reported by several formal and informal caregivers when observing older adults during VR use. Laughter [64,67,69], awe [59], positive mood [59,61,62,66], sensory stimulation, excitement, and surprise [59,61,62,66] were perceived across studies and across the immersion spectrum. A sense of enjoyment and happiness was mirrored across all levels of immersion through verbal feedback during use. However, in one study, this was captured when a person living with dementia became visibly moved and tearful [57,58,60-62,71]. Several studies observed older adults living with dementia express a positive response via song and dance through the incorporation of music into the VE [60,63,69]. The visual attractiveness and relevance of the VE have been reported to facilitate such responses [58,59,71].

Formal and informal caregivers reported VR as having a perceived positive impact on the mood, well-being, and everyday life of older adults living with dementia after VR use, as opposed to the above instances, which were during use. Family members and care staff reported lasting perceived improvements in cognition [65,66,68], memory [69], concentration [65], sustained attention [66], improved task organization [68], motivation [65,69], positive mood [60,62], reduced aggressive behaviors [62] and overall well-being [62] after the VR sessions. The specific time frame for the length of the sustained impact was not reported in the included studies. A longer-term impact was illustrated, where improvements were translated to increased engagement in activities of daily living and provided a sense of structure and purpose for older adults living with dementia. Such improvements include increased independence in dressing [65], increased motivation to exercise [62,65,69], and increased completion of kitchen tasks [68].

Caregivers also reported the negative impact of using VR. Caregivers observed confusion in one male living with dementia who wished to experience virtual content in the physical environment after VR use [66,70]. Caregivers also reported worsened behavioral and psychological symptoms of dementia [63,67], one incidence of increased hallucinations [67], and decreased mood [61,63] after use.

The sense of calm was a common thread across 7 studies both when actively interacting in the VE and when simply observing the visual and auditory content [59-62,66,67,71]. The ambient sounds and music in the VE were attributed to achieving this sense of calm by the care staff and older adults living with dementia [60,71]. Caregivers considered this “sedative effect” particularly applicable to older adults living with dementia who may become agitated. However, observational field notes in one study considered soothing audio or visual as inappropriate for those with apathy, and a more arousing audio and visual VE was suggested as an alternative [62,71].

Sensations related to simulator sickness, dizziness, and disorientation have been reported. Sickness during VR use was verbalized by older adults living with dementia in 1 of 7 fully immersive studies as feeling “sick” or having “a funny feeling” in their stomach [57]. Dizziness due to nonsynchronized hand and head movements was reported in one older adult living with dementia in one study [62]. Short-term disorientation was also observed when the headset was removed in 3 studies [61,67,70]. Two studies reported no sickness, dizziness, disorientation, or falls, which may be attributed to the semi-immersive and nonimmersive nature of these studies [65,68]. One fully immersive study highlighted the importance of a smooth transition back to physical space and adequate time spent in VR to avoid disorientation, sickness, and confusion [67]. Across the studies, the length of time regarding VR use varied, with no agreed length for optimal VR use [59,62,68,71].

#### Returning to Reality: Reflecting on the Virtual Experience

This theme explored reflections on the VR experience after returning to the physical space. Informal and formal caregivers’ changing attitudes and perceptions toward the technology after observing its use are also discussed.

For those who recalled the experience, people living with dementia exhibited varied responses to VR after the session. Experiences and perceptions existed along a continuum from satisfied to neutral and to dissatisfied. Some people living with dementia and caregivers expressed dissatisfaction and unwillingness to try VR again for several reasons including boredom [59,67], lack of stimulation [59,67], disinterest in habitual use [59,71], wanting to visit the physical space [60] and the childish nature of the design [71]. This was contrasted by several older adults living with dementia who described the experience as “marvelous” [59] and something they wanted to do “over and over” [59,61,67]. Neutrality of experience was also reported, where older adults living with dementia were impartial to VR use [59,67,71]. One older adult living with dementia in hospice care disclosed “it was alright; wasn’t good and wasn’t bad,” whereas another older adult living with dementia expressed “it’s a one-time experience, you don’t need it twice” [67] (p. 813). Residents in the hospice care facility study had a Functional Assessment Staging Tool score indicative of mid- to late-stage dementia, with a mean age of 85 years, which may account for this neutrality of experience.

Formal and informal caregivers reported a change in their attitude toward VR after observing older adults living with dementia using the system [61]. A care staff member in one inpatient setting reported the potential for multiuser VR to be overstimulating [63]. The care staff highlighted the need to value the age and physical functioning of older adults with dementia [59]. The variance in delivery and level of immersion across studies makes it difficult for formal caregivers to identify the optimal stage of dementia most suited to VR use [59,71].

### Summary of Synthesis

This QES illustrates 3 analytic constructs demonstrating key stakeholders’ experiences and perceptions of VR for older adults living with dementia throughout the VR journey. VR can be experienced positively, both momentarily and in the long term. However, the role of the facilitator is integral to achieving such outcomes and facilitating the step toward virtuality. The perception of VR varies before and after exposure to the technology, and the negative side effects of VR need to be acknowledged early in the implementation of such systems.

In addition to the thematic synthesis described in the findings section, recommendations from the primary included studies were extracted and consolidated to inform the future design of VR for older adults living with dementia. These recommendations are presented in Table 2 and categorized under different design element considerations.

**Table 2:** Consolidated virtual reality design recommendations.

Proposed design element	How to achieve the design element
Design for dynamic abilities and preferences [57-64,69-71]	Incorporate meaningful and varied scenes. Integrate items of personal relevance to stimulate conversation and engagement. Increase interactivity without increasing the difficulty. Design for passive and more active experiences based on the older adults’ needs and abilities. Facilitate items in the VE^a^ to come to the person instead of navigating to them. Include interactive elements, which can be adapted to increase or decrease difficulty if required. Incorporate multiple consistent experiences, which can be changed to maintain novelty. Avoid sensory overstimulation; present elements in the VE gradually and keep main interactable objects in the main field of view.
Everyday design [60-63,68,70]	Design tangible interfaces with familiar items to help with understanding and interactions. Include items in the VE, which can be associated with their semantic content.
Auditory elements [59-61,63,67]	Include personalized or personally relevant music or sounds. Use ambient sounds to accompany calming environments. Incorporate adaptable volume or sound control. Accommodate for hearing aids.
Visual elements [59,60,63,66,67,71]	Provide feedback for tasks and next steps. Integrate a reminder to appear before the session ending. Clearly highlight when the session has ended. Incorporate a range of interactable objects to avoid boredom. Include attractive visual content using bright and contrasting colors.
Social experiences [60,62,63]	Facilitate multiuser experiences. Include shared activities. Incorporate opportunities for social interaction.
Safety [57-60,62-64,68,70,71]	Administer an eligibility assessment before use. Identify accommodations before VR^b^ use such as glasses, hearing aids, standing or seated VR, etc. Provide adequate instruction for when VR is coming to an end and give ample time for the person to orientate themselves back to the physical space. Ensure facilitator presence during and immediately after VR use. Adequate frame rate and limit lagging to reduce simulator sickness. Provide a clear timeframe for use and incorporate breaks if required. Presence of a virtual agent in the VE to assist with interactions if required. Ensure a smooth transition back to the physical space.

^a^VE: virtual environment.

^b^VR: virtual reality.

## Discussion

### Principal Findings

This thematic synthesis identified 16 key findings exploring stakeholders’ experiences and perceptions of VR use by older adults living with dementia. This review reports the experiences and perceptions of older adults as self-reported by older adults living with dementia or through observations and reports of formal and informal caregivers. The findings were explored through the lens of 3 key analytic themes illustrating the VR journey. The experiences and perceptions of the spectrum of VR for older adults living with dementia have not been previously explored from a multistakeholder perspective or using QES. This review highlights the importance of conceptualizing VR use as a journey that has a clear beginning, middle, and endpoint. It is important that each stage of the “VR Journey” be sensitively introduced and facilitated with the dynamic needs of older adults living with dementia to the forefront.

Previous reviews had a limited focus on qualitative components, such as experiential, contextual, and implementation factors. This is the first known study to systematically synthesize qualitative evidence on key stakeholder experiences and perceptions across the spectrum of VR for older adults with dementia. The analysis and synthesis of the included studies suggested that VR was well-tolerated and could be a pleasant and positive experience for older adults living with dementia. Formal and informal caregivers also reported positive perceptions. This review yielded rich experiential data related to the positive effects of VR on the social and emotional well-being of older adults living with dementia, which is consistent with a similar literature review in this area [25]. The D'Cunha and Nguyen [25] review relates to broader literature reviews that explored VR use for people with sensory, cognitive, and physical health conditions who found VR to be tolerable and enjoyable [25,27,28,75] and elicited feelings of relaxation, adventure, and rejuvenation [75].

The barriers and facilitators of VR use were encapsulated in the analytical themes of this QES and add to the existing research in this area. It is evident that the novelty of VR and its associated preconceptions acted as a barrier to its use for some older adults living with dementia. Caregivers also exhibited preconceptions that affected their willingness to implement and facilitate VR in some care settings. Such barriers were counteracted through education, training, and adequate setup procedures, as evidenced in the findings. Akin to this, support and reassurance from others were found to dissipate some of the barriers associated with VR for older adults living with dementia. Facilitators of successful VR use are also highlighted in this QES and include acknowledging concerns and worries about initial use and the need for adequate setup and facilitation for formal and informal caregivers. The negative side effects and emotions, such as fear or anxiety, discussed in this review are also consistent with previous research [27] and are highlighted by Moreno et al [76] in a systematic review of people with neurocognitive disorders, including Alzheimer disease, with adverse effects reported by a minority of participants. The Moreno et al [76] findings suggest that adverse effects are not exclusive to this population but are universal and should be monitored as with any user group [76]. Moreover, previous research has acknowledged that the diagnosis of dementia is not a barrier to the use of VR systems. However, procedures must be in place to adequately step into virtuality [77]. A meta-analysis undertaken by Kim et al [26] pointed to the need for adaptive systems and, in particular, methodology type and interaction technique that account for the sensory needs of people living with dementia [26]. Future research should consider and address these barriers and facilitators to ensure positive VR experiences for all stakeholders.

The setting also appeared to be a facilitator for VR use in several of the included studies. Baker et al [78] and Miller et al [79] explored VR use in residential and community facilities and suggested that environments with staff facilitation, Internet connectivity, and physical space may provide additional support and encouragement in terms of VR setup and other feasibility issues that may not be as apparent in the home environment. They also suggested that using VR in a home environment may be more convenient and comfortable and elicit different experiences of using the technology, particularly at the preparatory and initial stages of use [78,79].

This review provides evidence of the need to explore the importance of “in the moment” experiences versus long-term sustained outcomes. At present, evaluations have been cited as the most authentic form of data collection and reflect how a person with dementia feels at a given time [80]. VR may provide a means of being in the moment and help frame the lived experiences of people living with dementia [81]. Consistent with the findings of this review, previous empirical research [82,83] has acknowledged the lack of apparent lasting outcomes associated with VR use and stated that a lack of such outcomes should not devalue the overall experience [82,83]. Instead, the focus should be shifted to the philosophy of doing so for the moment [81,82,84,85]. Indeed, health and social care professionals are encouraged to meet people living with dementia “in” the moment, and focus on reporting the value of the present, here, and now experiences [81,84,86].

Despite VR systems being designed for single users, the social element of VR appears as a recurring thread across the included studies. It is evidenced in the literature [87-90] that designing for social connectedness is especially pertinent for people living with dementia, as they may have a reduced ability to socialize and maintain relationships. The importance of maintaining social connections was vital during COVID-19, as people living with dementia were unable to physically meet others [91-93]. This review mirrors the wider literature highlighting digital technology use as a ticket-to-talk for people living with dementia [94,95]. Syed-Abdul et al [96], and Lin et al [97] acknowledged the potential for multiuser spaces for older people living with dementia who can interact in the VE while simultaneously advocating for VR, which facilitates group participation to promote social interactions. This review supports the call for more Social VR spaces for older adults living with dementia and provides useful guidance on how this can be incorporated into future VR spaces.

The findings of this QES study highlight the value of designing VR spaces to facilitate dynamic interactions. In a previous meta-analysis, Kim et al [26] acknowledged that diverse VR functions may be perceived as too complex for older adults. This QES demonstrated that positive interactions and feelings of empowerment may be attributed to the hardware used. Furthermore, the dynamic interactions and experiences of older adults living with dementia may be attributed to the variance in hardware and software systems. The importance of considering which VR hardware is best suited for older adults living with dementia is also highlighted in this QES. Some of the included studies used realistic 360° video, which Yeo et al [98] contended may not be a representative form of the natural environment, as video content filming is completed in advance, and this limits the user’s agency over where to go. In contrast, other studies have used computer-generated VEs, which may afford more interaction, agency, and a more dynamic experience [99]. Furthermore, Strong [28] suggested that the difference between these modes of delivery may be an important factor in assessing one’s sense of immersion and presence.

This QES highlights a lack of stakeholder involvement, as most of the included studies failed to report on the VR design process. Akin to the role of stakeholders in the design process, older adults living with dementia must have their voices heard when using and evaluating VR. Most of the included studies emphasized a proxy means of data collection from health care professionals and family members rather than collecting data from the target main user, that is, older adults living with dementia. Similar to Chopra and Dixon [100], our findings advocate for people living with dementia and critical stakeholders to inform the design of VR from the outset, transcending their role beyond that of the end user of the technology. Although stakeholders may hold their own expectations and interests in the research, their views may not be consistent with, and may suppress and replace, the voice of the person with dementia [101-106]. Some advocates for the prioritization of the voices of people living with dementia use the views of secondary stakeholders as a supplement, which is reflected in this QES [101]. However, there is a need to consciously involve people living with dementia through innovative and strength-based methods and techniques such as the CoRTE framework, which comprises 4 main domains when conducting interviews with people living with dementia: gaining consent, maximizing responses, telling the story, and ending on a high [106-110].

The QES provides a novel overview of the spectrum of willingness to try VR in the context of various levels of immersion. Rose et al [61] found that people living with early-stage dementia reported boredom or a lack of relevance when using the VR system [61]. The question then arises as to the preference for personalized VR experiences over generic VR experiences. Hodge et al [60] and Hodge and Morrissey [86] suggested that personalized VR experiences may be more meaningful, whereas generic VR experiences may be cost-effective and offer increased transferability. A dilemma is then posed for designers, as they are faced with this dichotomy of personalized or personal relevance versus transferability. One solution may be to design for personal and contextual relevance, whereby VEs achieve a middle ground, affording older adults living with dementia the opportunity to use artifacts in the VE as a scaffold for meaningful experiences [78].

### Limitations of the Review

This review adopted a broad approach to defining VR, which makes it difficult to compare across the spectrum of immersion. This is also true for the diverse implementation of VR, whereby some systems were part of an intervention, while others were once-off recreational experiences. Consistent with other reviews in the VR and gerontology landscapes, there is a lack of consistency in defining VR [111]. Thus, it was challenging to adequately identify and assess eligibility for inclusion. Search strings included the term “virtual environment” and “virtual reality” to ensure both terms, (often referred to along the reality-virtuality continuum) were utilized [111]. It should be noted that the review did not aim to identify all literature on the topic but to identify those papers “with characteristics relevant to the phenomenon being studied, not statistical representativeness” [112]. The authors acknowledge that the systematic search was completed in October 2020; thus, relevant research published after this date may be excluded from this review. The methodological limitations of the individual included studies were acknowledged in the assessment of confidence in the findings using the GRADE-CERQual.

### Implications for Practice and Future Research

This review aimed to complete a systematic QES that used best practice in assessing confidence in the research findings, an approach that may complement quantitative reviews on the use of VR for older adults living with dementia, and provide insights into the contextual factors related to its use [32,113]. This QES highlights the need for additional qualitative research on VR use in older adults living with dementia. It also stipulates the need for design recommendations to inform future design and research. Additional reporting on the design process, ethical procedures, data collection methods, and researcher reflexivity is warranted. Further research on VR use for sociability outcomes is required, which should mirror current digital technology and Social VR research for the general older adult population [57,78,114-117]. These seminal works may guide future VR use for older adults living with dementia and may be adapted to accommodate the needs of this dynamic population.

Clinicians refer to the current clinical state of VR as the “Wild West” [118] because there is an apparent lack of guidance on the length and frequency of VR for people living with dementia, formal and informal caregivers, and facilitators of VR and HCI researchers [26,28,118-120]. The broader HCI literature suggests a need for coherent design knowledge and frameworks to ensure what Tabbaa, Ang [120] term as “effective, enriched and meaningful” VR spaces. The findings of this review provide several recommendations for future research (Table 2). Furthermore, the authors advocate for a designated, trained facilitator with a protected time for VR use and highlight the integral role of facilitators in encouraging VR use.

### Conclusions

Considering that there is currently no cure for dementia, strategies to support the psychosocial needs of older adults living with this condition are crucial. The use of VR for older adults living with dementia is a growing area, and this review suggests that it is a promising addition to dementia care. The QES provides recommendations for future VR design and implementation. The potential positive effects of VR use include a sense of connection, empowerment, immersion, calmness, and enlivenment. However, the potential negative implications of VR use must be considered, such as simulator sickness, fatigue, and disorientation. Moreover, the optimal hardware, software, and dosage best suited to older adults living with dementia remain ambiguous and warrant further guidance. On the basis of this QES, VR provides a means of facilitating sociability outcomes despite not being designed for this specific focus. Consequently, the authors suggest future exploration of Social VR for older adults living with dementia to facilitate social connectedness through multiuser VR spaces.

## Appendix

Multimedia Appendix 1 Search strategy.

Multimedia Appendix 2 Screening log.

Multimedia Appendix 3 Inclusion and exclusion criteria.

Multimedia Appendix 4 Characteristics of included studies table.

Multimedia Appendix 5 Methodological limitations.

Multimedia Appendix 6 Summary of qualitative findings and confidence rating.

Multimedia Appendix 7 Confidence in the evidence from reviews of qualitative research (GRADE-CERQual) full evidence profile.

Multimedia Appendix 8 Supporting quotations.

## Abbreviations

GRADE-CERQual: confidence in the evidence from reviews of qualitative research
HCI: human-computer interaction
HMD: head-mounted display
MeSH: medical subject headings
OT: occupational therapy
PRISMA: Preferred Reporting Item for Systematic Reviews and Meta-Analyses
QES: qualitative evidence synthesis
RACF: Residential Aged Care Facility
RETREAT: Review question-Epistemology-Time or Timescale-Resources-Expertise-Audience and purpose-Type of Data
VE: virtual environment
VR: virtual reality
