# Supplementary material for: Key Stakeholders’ Experiences and Perceptions of Virtual Reality for Older Adults Living With Dementia: Systematic Review and Thematic Synthesis
Source: JMIR Serious Games. 2022 Dec 23;10(4):e37228. doi: 10.2196/37228 (PMC9823606; doi:10.2196/37228)
Supplement: Multimedia Appendix 1 [file games_v10i4e37228_app1.docx]

**Multimedia Appendix 1: MEDLINE Search Strategy**

| 1. Dementia/ or Frontotemporal Dementia/ or Dementia, Vascular/,  2. Alzheimer Disease/,  3. Lewy Bodies/,  4. Huntington Disease/,  5. Creutzfeldt-Jakob Syndrome/,  6. "Pick Disease of the Brain"/,  7. dementia.tw.,  8. vascular dementia.tw.,  9. frontotemporal.tw.,  10. lewy bod*.tw.,  11. huntington*.tw.,  12. pick*.tw.,  13. creutzfeldt-jacob.tw.,  14. alzheimer*.tw.,  15. ((memory or mental or cognitive) adj3 (decline or impair* or insufficien* or complain* or disorder* or loss or deteriorat*)).tw.,  16. Virtual Reality/,  17. VR.tw.,  18. virtual realit*.tw.,  19. (virtual adj3 realit* technolog*).tw.,  20. virtual environment*.tw.,  21. (virtual adj3 environment*).tw.,  22. immersive.tw.,  23. non-immersive.tw.,  24. semi-immersive.tw.,  25. audiovisual.tw.,  26. experience*.tw.,  27. percept*.tw.,  28. perspect*.tw.,  29. view*.tw.,  30. opinion*.tw.,  31. attitude*.tw.,  32. tolera*.tw.,  33. feasib*.tw.,  34. barrier*.tw.,  35. facilitat*.tw.,  36. accept*.tw.,  37. usab*.tw.,  38. 26 or 27 or 28 or 29 or 30 or 31 or 32 or 33 or 34 or 35 or 36 or 37,  39. 1 or 2 or 3 or 4 or 5 or 6 or 7 or 8 or 9 or 10 or 11 or 12 or 13 or 14 or 15,  40. 16 or 17 or 18 or 19 or 20 or 21 or 22 or 23 or 24 or 25,  41. 38 and 39 and 40, |
| --- |
