# Supplementary material for: Key Stakeholders’ Experiences and Perceptions of Virtual Reality for Older Adults Living With Dementia: Systematic Review and Thematic Synthesis
Source: JMIR Serious Games. 2022 Dec 23;10(4):e37228. doi: 10.2196/37228 (PMC9823606; doi:10.2196/37228)
Supplement: Multimedia Appendix 2 [file games_v10i4e37228_app2.docx]

**Multimedia Appendix 2: Screening Log**

**Database Duplicate Log**

| Data Source | | Duplicates Results | | |
| --- | --- | --- | --- | --- |
| Database | **Items  found** | **Inner  dups** | **Ext  dups** | **New** |
| MEDLINE | 157 | 0 | 0 | 157 |
| PsychINFO | 261 | 1 | 81 | 179 |
| CINAHL | 77 | 5 | 33 | 39 |
| AgeLine | 33 | 1 | 17 | 15 |
| Scopus | 663 | 6 | 85 | 572 |
| Compendex | 447 | 9 | 351 | 87 |

| Items found | Inner dups | Ext dups | New |
| --- | --- | --- | --- |
| 1638 | 22 | 567 | **1049** |
|  |  |  |  |
|  |  |  |  |
| Total duplicates: 589  Total for screening: 1049 | | | |

**Screening Log**

| Database Title and Abstract | | |
| --- | --- | --- |
| Total Screened | 100% | 1049 |
|  |  |  |
| Pre-Conflict Resolution |  |  |
|  | Included: | 54 |
|  | Excluded: | 965 |
|  | Conflict: | 30 |
|  |  |  |
| Post-Conflict Resolution |  |  |
|  | Included: | 67 |
|  | Excluded: | 982 |
|  |  |  |
| Forward and Backward Title and Abstract Screening | | |
| Total Screened | 100% | 40 |
| Pre- Conflict Resolution | Included: | 16 |
|  | Excluded: | 20 |
|  | Maybe: | 4 |
|  | Undecided: | 0 |
| Post- Conflict Resolution | Included | 18 |
|  | Excluded | 23 |
|  | | |

| Database Full Text Screening | | |
| --- | --- | --- |
| Full-Text Screening Deadline: | 30/10/20 |  |
| Pre- Conflict Resolution |  |  |
|  | Included: | 9 |
|  | Excluded: | 37 |
|  | Conflicts: | 6 |
|  | Undecided | 1 |
|  | Maybe: | 14 |
| Post- Conflict Resolution (i) |  |  |
| 11/12/2020 | Included: | **12** |
|  | Excluded: | 53 |
|  | Maybe: | 1 |
|  | Undecided | 1 |
|  |  |  |
| Post-Conflict Resolution (ii) | Included: | **12** |
| *AF and DH discussed remaining conflicts with CH* | Excluded | 55 |
|  |  |  |
| Forward and Backward Full Text Searching | | |
| Pre-Conflict Resolution | Included: | 3 |
|  | Excluded: | 6 |
|  | Maybe: | 8 |
| Post- Conflict Resolution | Included | **3** |
|  | Excluded | 15 |
|  |  |  |
| Total Included for Data Extraction | | |
| 15 reports of 14 studies | | |
