# Supplementary material for: Key Stakeholders’ Experiences and Perceptions of Virtual Reality for Older Adults Living With Dementia: Systematic Review and Thematic Synthesis
Source: JMIR Serious Games. 2022 Dec 23;10(4):e37228. doi: 10.2196/37228 (PMC9823606; doi:10.2196/37228)
Supplement: Multimedia Appendix 3 [file games_v10i4e37228_app3.docx]

**Multimedia Appendix 3: Inclusion and Exclusion Criteria**

| **Inclusion** | **Exclusion** |
| --- | --- |
| - Use of VR   - Non-immersive (low)   - Semi-immersive (moderate)   - Fully immersive (high)   - Classification as per Miller et al, 2016 - Inclusion of stakeholders in the field of dementia care   - People living with dementia (any type or severity)/ clinical diagnosis, GP diagnosis or on dementia specific medication   - Mean age over 60   - Family support person (informal caregiver)   - Health and Social Care Professionals e.g Nursing, Social worker, Physiotherapist, Occupational Therapist, GP, Gerontologist, Psychologist   - Those involved in the development or facilitation of VR   - Others (can be discussed if others arise during conflict resolution) - Use of qualitative data collection which address experiences and perceptions of VR   - Their perspective may incorporate their opinions, attitudes, and evaluations (including acceptance and satisfaction) (Ryan et al, 2018).   - Their experiences may relate to emotions, physical sensations (e.g. pain, discomfort), psychological factors (e.g. stress, mood), and pragmatic factors (e.g. routine activities) (Ryan et al, 2018). | **Exclusion:**   - No inclusion of people living with dementia or key stakeholders - Unable to extract subset data for people living with dementia - No VR used: exclude augmented reality - Quantitative Studies - Non-peer reviewed articles - Literature/Systematic reviews |

Miller H. L, Bugnariu N. L. Level of immersion in virtual environments impacts the ability to assess and teach social skills in autism spectrum disorder. Cyberpsychology, Behavior and Social Networking. 2016;19(4):246–256. doi:10.1089/cyber.2014.0682

Ryan C, Hesselgreaves H, Wu O, Paul J, Dixon-Hughes J, Moss J. G. Protocol for a systematic review and thematic synthesis of patient experiences of central venous access devices in anti-cancer treatment. Systematic Reviews.2018; 7(1): 61. doi:10.1186/s13643-018-0721-x.
