# Supplementary material for: Key Stakeholders’ Experiences and Perceptions of Virtual Reality for Older Adults Living With Dementia: Systematic Review and Thematic Synthesis
Source: JMIR Serious Games. 2022 Dec 23;10(4):e37228. doi: 10.2196/37228 (PMC9823606; doi:10.2196/37228)
Supplement: Multimedia Appendix 4 [file games_v10i4e37228_app4.docx]

| Multimedia Appendix 4: Characteristics of included studies | | | | | | | | | |
| --- | --- | --- | --- | --- | --- | --- | --- | --- | --- |
| Citation | Country | Study setting | Research Design and Methods of Data Collection | Participant Information | Stage of Dementia (formal or informal Diagnosis) | Level of technology Experience | Level of Immersion, Hardware and Software | Duration of VR Experience | Data Analysis |
| Baker et al, 2020  [57] | Australia | RACF | Qualitative multi-method design  Interviews with residents and staff, visual rating instrument, field observations | One older adult living dementia  Sex: Male  Age: 88 years  Five staff members  Sex: Not specified  Age: Not specified | “Formal dementia diagnosis” | Older adult living with dementia experience of VR: None  Staff experience of VR:  None:3  Had seen it:1  Had seen and used it: 1 | Fully immersive  Hardware:  Oculus Rift HMD and controller  Software:  First Contact and Google Earth VR | Four, 60 minute VR sessions | Burnard's (1991) approach to thematic content analysis |
| D'Cunha et al, 2020  [58] | Australia | RACF | Mixed-method randomised control trial, crossover, feasibility study  Semi-structured interviews with older adults living with dementia and activity manager | 10 Residents living with dementia  Sex: 8  Female, 2 Male  Age: Average age 86.1 (±8.06) years.  Age range: 74-95 years  1 RACF activity manager  Sex: Male  Age: Not specified | MoCA score:  5/30-21/30  Average score of 12.2 (±4.69) | Not specified | Semi-immersive  Hardware:  GoPro Hero 7  Projector screen,  Body Charger ® GB3030 UBE  Software:  Pre-recorded content | 25 minutes | Content analysis (Graneheim & Lundman, 2004) |
| Feng et al, 2018  [66] | Netherlands | RACF | User evaluation study  Semi-structured interviews | 15 residents  Sex: 12  Female 12, Male 3  Age:  Mean:87 SD :5  Age range:79-97 years  Four family members of residents  Sex:Male:3  Female:1  Age:  Mean: 47 years SD:4  Two professional caregivers  Sex: Female  Age: Not specified  Mean:36years SD:6 | Dementia type:  Alzheimer: 6  Vascular: 3  Mixed: 6  Stage:  Mild:4  Moderate:6  Severe:5 | Not specified | Non-immersive  Hardware:  87-inch Screen  with water pump as interaction/controller  Software:  Computer connected to Arduino | Always on to interact using the water pump as the controller. | Thematic analysis |
| Ferguson et al, 2020  [67] | USA | Hospice | Qualitative descriptive design  Semi-structured interviews with older adults living with dementia | 25 People living with dementia  Sex: 22 Female, 3 Male  Age:  Mean: 85 years SD:8.9  Range 68-103 years | Dementia type:  Alzheimer:11  Vascular: 6  Mixed Alzheimer and vascular: 5  Multiple sclerosis dementia: 1  Not specified dementia : 2 | Not specified | Fully immersive  Hardware:  MirageSolo HMD with Daydream Business Edition  Software:  360-degree video and YouTube VR | 3.5-minute video looped up to 12 times  30-minute limit | Content Analysis |
| Foloppe et al, 2018  [68] | France | Community setting (Participant’s home) | A within-single case study design  Behavioural observation and comments made by the patient about the task and the usability of the system were noted as qualitative feedback | One person living with dementia  Sex: Female  Age: 79 | Alzheimer’s dementia | Not specified | Non-immersive  Hardware:  Laptop computer and mouse.  Software:  Unity 3D | Four, 60 minute tasks | Not specified |
| Hodge et al, 2018  [60] | UK | Bluebell Grove: a registered local charity for people living with dementia | Qualitative design study  Researcher field notes, semi-structured interviews with key participants | Four people living with dementia  Three Partners/Family members  Sex: Male 3, Female 4  Age Range: 51-84 years | Type:  All participants had Alzheimer’s  Stage:  One participant cited as having mild dementia | Not specified | Fully immersive  Hardware:  HMD  Google Cardboard  Software:  Unity 3D  Scripted in C# | Not specified | Thematic Analysis (Braun & Clarke, 2006) |
| Matsangidou et al, 2020  [70] | Cyprus | Psychiatric hospital | Mixed-method pilot study  Researcher observations | Study 2  10 people living with dementia  Sex: 5 Female, 5 Male  Age: 75 to 85 years  M: 78.90,  SD: 3.81  Study 3  10 people living with dementia  Sex: 5 Female, 5 Male  Age: 65 to 90 years  M: 80.10  SD: 7.11 | Study 2  Type:  Alzheimer’s disease: 10  Stage:  Moderate to severe dementia.  GDS rating was 4.80 (SD: .92)  Study 3:  Type:  Alzheimer’s disease: 6  Not specified dementia: 4  Stage:  Moderate to severe  GDS mean was 4.71 (SD: .95) | Not specified | Fully immersive  Hardware:  Oculus Rift CV1 VR1 (HMD),  Oculus Touch Controllers  Software:  Unity3D,  3Dmodels created using Maya3 | Study 3: The maximum duration 20 minutes | Content Analysis |
| McEwen et al, 2014  [65] | Canada | Outpatient setting | Single-subject feasibility study  Open-ended interviews with family and staff members. | One older adult living with dementia  Sex: Male  Age: 78 years  One caregiver also interviewed  Unspecified number of family members  Age: Not specified  Sex: Not specified | Type:  Vascular dementia  Stage:  MoCA score 12/30 | Not specified | Semi-immersive  Hardware:  50-inch TV screen  Software:  Interactive rehabilitation exercise (IREX) software | 2 weeks, 5 days a week  Each session lasted approximately 1 h with an average of 25 min of VR exercise time | Themes extracted |
| Moyle et al, 2018  [59] | Australia | RACF | Mixed-method pilot study  Video recorded observations and interviews | 10 people living with dementia over 60 years of age  Sex: 7 Female, 3 Male  Age:  Mean: 89 SD:4.97  10 family members  Sex: 6 Female, 2 Male, 1 Undisclosed  Age:  35-44 :1  45-54 :3  55-64: 3  65-74:1  75-84:1  Undisclosed:1  9 care staff  Sex : 9 Female  Age:  35-44: 3  45-54:3  55-64:1  Greater than or equal to 65: 1 Undisclosed:1 | People living with dementia:  Type:  Alzheimer’s: 7 Undisclosed type: 3  Stage:  PAS score range: 7.35-20  Average score: 13.21 | Not specified | Semi-immersive  Hardware:  Large, projected interactive screen,  Microsoft Kinect motion sensors  Software:  Video game technology, involving vivid graphics and motion sensors | Maximum of 15 minutes | Thematic Analysis |
| Park, 2019  [71] | South Korea | RACF | Mixed-methods study  Video observations, interviews with staff and older adults living with dementia. | 30 older adults living with dementia  Sex: majority female  Age:  Mean 70.4 years. Range: 59-83 years.  Average age of males: 69.18  Average age of females: 71.15  15 care staff  Sex: Undisclosed  Age: Undisclosed | Type:  Alzheimer’s disease or other form of dementia  Stage:  PAS mean score: 14.25 | Not specified | Fully immersive  Hardware:  Oculus,  Microsoft Kinect,  Leap motion sensor  Software:  Unity 3D,  Windows | 3 days a week  15 minutes | Not specified |
| Rose et al, 2019  [61] | UK | Inpatient psychiatric care setting | Mixed-method feasibility study  Observations and Semi-structured interviews | Eight people living with dementia  Sex: 2 Female, 6 Male  Age:  Mean: 69.63 years  Range: 41-88 years  16 caregivers  Sex: Not specified  Age: Not specified | Type:  Alzheimer’s: 2  Dementia with huntington:2  Vascular:1  Frontotemporal dementia: 1  Not specified dementia: 2 | Older adults living with dementia did not report to have previously used HMD-VR. | Fully immersive  Hardware:  Samsung Gear VR with Samsung Galaxy S6 mobile phone  Software:  360-degree videos | 15 minutes maximum | Thematic analysis (Braun & Clarke, 2006) |
| Siriaraya and Ang 2014  [63] | UK | Two care home in South-East England | Mixed-methods study  Observations, focus groups and interviews | Approx. 20 Care home residents living with dementia  Sex: Not specified  Age: 80+ years  Six carers/activities facilitators  Two are home managers  Sex: Not specified  Age: Not specified | All had “some form of dementia” | Little experience of VR (not actually measured) | Semi-immersive  Hardware:  Projector,  Microsoft Kinect  Software:  Unity3D | Not specified | Thematic analysis |
| Siriaraya et al., 2017  [64] | UK | RACF | Case study  Observation sessions. The first session involved three staff members, eight care home residents and three volunteers. The second session involved two staff members, four clients and one volunteer. | Session 1:  Eight people living with dementia  Sex: Not specified  Age: 80+ years  Three staff members  Sex: Not specified  Age: Not specified  Three volunteers  Sex: Not specified  Age: Not specified  Session 2:  Four people living with dementia  Sex: Not specified  Age: 80+ years  Two staff members  Sex: Not specified  Age: Not specified  One volunteer  Sex: Not specified  Age: Not specified | All had “Some form of dementia” | Not specified | Semi-immersive  Hardware:  Projector,  Microsoft Kinect sensor,  Laptop,  Replica Plants  Software:  Not specified | 20-25 minutes | Not specified |
| Tabbaa et al, 2019  [62] | UK | Locked psychiatric hospital in the UK that specialises in progressive neurological conditions, including dementia. | Mixed-methods study  Interviews, observations | Eight people living with dementia  Sex: 2 Female, 6 Male  Age:  Mean 69.63  Range = 41-88  16 caregivers  Sex: Not specified  Age: Not specified | Alzheimer’s: 2  Not specified dementia:2  Dementia with huntington:2  Vascular: 1  Frontotemporal: 1 | Not specified | Fully immersive  Hardware:  Samsung Gear VR1 HMD paired with a Samsung Galaxy S6 mobile phone  Software:  360-degree Video | Maximum of 15 minutes | Thematic Analysis (Braun & Clarke, 2006) |
| Unbehaun et al, 2020  [69] | Germany | Day-care centres, care-facilities and domestic households | Qualitative methodology, 4-month evaluation study  Interviews with participants, relatives and professional caregivers | 53 people living with early-mid-stage dementia  Sex: Not specified  Age:  Mean:78 SD:8  Range: 53-94  25 caregivers and relatives  Sex: 87% Female  Age:  Mean: 66 SD:4  Range: 34-97 | Type:  ICD 10-diagnosed dementia: Alzheimer type or vascular dementia  Stage:  Early-mid stage dementia | Not specified | Non-immersive  Hardware:  TV screen,  Microsoft Kinect, PlayStation 3Buzzer  Software:  Unity 3D Game Engine scripted using C# | 20-25 minutes daily | Thematic analysis (Braun & Clarke, 2006) |
