# Supplementary material for: Key Stakeholders’ Experiences and Perceptions of Virtual Reality for Older Adults Living With Dementia: Systematic Review and Thematic Synthesis
Source: JMIR Serious Games. 2022 Dec 23;10(4):e37228. doi: 10.2196/37228 (PMC9823606; doi:10.2196/37228)
Supplement: Multimedia Appendix 5 [file games_v10i4e37228_app5.docx]

| **Multimedia Appendix 5: Methodological limitations of the included studies** | | | | | | | | | | |
| --- | --- | --- | --- | --- | --- | --- | --- | --- | --- | --- |
| **Study ID** | **Was there a clear statement of the aims of the research?** | **Is a qualitative methodology appropriate?** | **Was the research design appropriate to address the aims of the research?** | **Was the recruitment strategy appropriate to the aims of the research?** | **Was the data collected in a way that addressed the research issue?** | **Has the relationship between researcher and participants been adequately considered?** | **Have ethical issues been taken into consideration?** | **Was the data analysis sufficiently rigorous?** | **Is there a clear statement of findings?** | **Overall assessment of methodological limitations** |
| Baker et al, 2020  [57] | Yes | Yes | Yes | Yes | Yes | Can’t Tell | Yes | Yes | Yes | No or very minor concerns. Unclear statement of researcher reflexivity. |
| D’Cunha et al, 2020  [58] | Yes | Yes | Yes | Yes | Yes | No | Yes | Yes | Yes | No or very minor concerns. No statement of researcher reflexivity. |
| Feng et al, 2018  [66] | Yes | Yes | Yes | No | Yes | No | No | Yes | Yes | Moderate concerns: regarding how data was collection (informed consent, capacity assessment etc.), to statement of reflexivity, recruitment procedures also omitted/unclear |
| Ferguson et al, 2020  [67] | Yes | Yes | Yes | Yes | Yes | No | Yes | Yes | Yes | No or very minor concerns: concern regarding reflexivity and positionality of the researcher. |
| Foloppe et al, 2018  [68] | Yes | Can’t Tell | Can’t Tell | Yes | Can’t Tell | No | Can’t Tell | No | Yes | Serious concerns: lack of description of the qualitative methods used, data collection procedures, researcher reflexivity, ethical considerations, data analysis procedures. |
| Hodge et al, 2018  [60] | Yes | Yes | Yes | Yes | Yes | Yes | Yes | Yes | Yes | No or very minor concerns: rich and robust account of the research with a clear statement of reflexivity. |
| Matsangidou et al, 2020  [70] | Yes | Yes | Yes | Yes | Yes | No | No | Yes | Yes | Moderate concerns: lack of detail on researcher reflexivity and ethical considerations. |
| McEwen et al, 2014  [65] | Yes | Yes | Yes | Yes | Can’t Tell | No | Yes | No | Yes | Moderate concerns: lack of detail regarding researcher reflexivity, data collection procedures and data analysis approach. |
| Moyle et al, 2018  [59] | Yes | Yes | Yes | Yes | Yes | No | Yes | Yes | Yes | No or very minor concerns: lack of detail on researcher reflexivity. |
| Park 2019  [71] | Yes | Yes | Yes | Yes | Yes | No | No | No | Yes | Serious concerns: ethical procedures omitted, no data analysis approach specified, no statement of reflexivity. |
| Rose et al, 2019  [61] | Yes | Yes | Yes | Yes | Yes | No | Yes | Yes | Yes | No or very minor concerns: Lack of researcher reflexivity statement. |
| Siriaraya & Ang 2014  [63] | Yes | Yes | Yes | No | Yes | Yes | Can’t Tell | Yes | Can’t Tell | Moderate concerns: lack of detail on recruitment strategies, ethical procedures employed and limited statement of findings presented. |
| Siriaraya et al, 2017  [64] | Yes | Can’t Tell | Yes | Can’t Tell | Can’t Tell | No | No | No | Can’t Tell | Serious concerns: lack of detail of design, data collection, analysis, ethical procedures; lack of researcher reflexivity |
| Tabbaa et al, 2019  [62] | Yes | Yes | Yes | Yes | Yes | No | Yes | Yes | Yes | No or very minor concerns: Lack of researcher reflexivity statement. |
| Unbehaun et al,2020  [69] | Yes | Yes | Yes | Yes | Yes | No | Yes | Yes | Yes | No or very minor concerns: Lack of researcher reflexivity statement. |
