# Supplementary material for: Key Stakeholders’ Experiences and Perceptions of Virtual Reality for Older Adults Living With Dementia: Systematic Review and Thematic Synthesis
Source: JMIR Serious Games. 2022 Dec 23;10(4):e37228. doi: 10.2196/37228 (PMC9823606; doi:10.2196/37228)
Supplement: Multimedia Appendix 6 [file games_v10i4e37228_app6.docx]

Multimedia Appendix 6. Summary of qualitative findings and confidence rating.

|  | Studies contributing to the review finding | CERQual assessment of confidence in the evidence |
| --- | --- | --- |
| Stepping into virtuality |  |  |
| *Stepping into the unknown* |  |  |
| Finding 1: Older adults living with dementia and caregivers may have concerns regarding the fear and physical discomfort of VR systems. | [57, 59-61, 68, 70] | High confidence |
| Finding 2: Education, training and set-up procedures for older adults living with dementia and caregivers can be useful for assessing eligibility, providing reassurance, safety and highlight potential benefits of VR. | [57-60, 63, 68, 71] | High Confidence |
| *Supporting the step into virtuality* |  |  |
| Finding 3: The continual presence of the caregiver/family member during the VR experience can provide encouragement, motivation and reassurance for older adults living with dementia to use VR. | [58-60, 69-71] | Moderate Confidence |
| Finding 4: Support and facilitation must be adapted to the needs of older adults with dementia as they are engaging in VR. This may be through verbal praise and encouragement, physical readjustment or assistance with the system or adapting the virtual environment to suit one’s needs. | [57-60, 63, 64, 69-71] | Moderate Confidence |
| Escape to virtuality |  |  |
| *An immersive world* |  |  |
| Finding 5: Older adults living with dementia can experience a sense of immersion, presence or embodiment in the virtual environment. | [58, 61-63, 66, 71] | Moderate Confidence |
| *Unlocking and maintaining connections* |  |  |
| Finding 6: For older adults living with dementia, VR can provide connections to broader experiences beyond where they currently reside. | [58, 60-63, 69, 71] | High Confidence |
| Finding 7: VR can provide a means of unlocking memories and connections to the past for older adults living with dementia. | [58-64, 66, 69, 70] | Moderate confidence |
| Finding 8: VR can provide an opportunity for shared experiences and can enhance the social connection and engagement of older adults living with dementia. | [58, 60-64, 66, 67, 69, 71] | Moderate confidence |
| *Interaction and empowerment* |  |  |
| Finding 9: When the level of interaction is suited to the abilities of older adults living with dementia they may achieve a sense of agency, empowerment and control in the VE. However, when the level of interaction is not suited the opposite may be experienced. | [58-60, 62, 63, 66, 69, 71] | Moderate confidence |
| Finding 10: Older adults living with dementia may demonstrate dynamic use of VR. This includes varying length of use, levels of interaction, difficulty and tolerability of the system. | [57-71] | Moderate confidence |
| *Physical, cognitive and affective responses* |  |  |
| Finding 11: VR can provide an ‘enlivening’ experience for older adults living with dementia. Enjoyment, happiness, laughter, awe, positive mood, sensory stimulation, excitement and surprise may be exhibited when using VR. | [57-64, 66, 67, 69-71] | Moderate confidence |
| Finding 12:  VR may provide a soothing and calming experience for some older adults living with dementia. | [59-62, 66, 67, 71] | High Confidence |
| Finding 13: VR use may result in negative emotions and sensations for older adults living with dementia including: dizziness, disorientation, BPSD and fatigue. | [57-59, 61-63, 65-68, 70, 71] | Moderate Confidence |
| Finding 14: VR effects may be translated into the daily lives and routine of older adults living with dementia. Improvements in cognition, memory, concentration, sustained attention, improved task organization, motivation, mood and overall wellbeing may be perceived after VR sessions. | [59, 61-63, 65, 66, 68, 69] | High Confidence |
| Returning to reality: Reflecting on the virtual experience |  |  |
| Finding 15: Older adults living with dementia can exhibit a spectrum of willingness to try VR again indicated by positive, neutral, and negative comments. Older adults with dementia can share their positive experiences and anticipation with their peers and community. | [57-61, 64-67, 69, 71] | Moderate confidence |
| Finding 16: Formal and informal caregivers may identify a change in their own attitude toward VR and may exhibit new understanding of the capabilities of older adults living with dementia after observing older adults living with dementia use the system. | [59-63, 69, 71] | High Confidence |
