# Supplementary material for: Key Stakeholders’ Experiences and Perceptions of Virtual Reality for Older Adults Living With Dementia: Systematic Review and Thematic Synthesis
Source: JMIR Serious Games. 2022 Dec 23;10(4):e37228. doi: 10.2196/37228 (PMC9823606; doi:10.2196/37228)
Supplement: Multimedia Appendix 7 [file games_v10i4e37228_app7.docx]

| **Multimedia Appendix 6: GRADE CERQual Full Evidence Profile** | | | | | | | |
| --- | --- | --- | --- | --- | --- | --- | --- |
| Summary of review finding | Reports of studies contributing to the review finding | Methodological limitations | Coherence | Relevance* | Adequacy | CERQual assessment of confidence in the evidence | Explanation of CERQual assessment |
| Finding 1: Older adults living with dementia and caregivers may have concerns regarding the fear and physical discomfort of VR systems. | [57, 59-61, 68, 70] | *No or very minor concerns*  No or very minor concerns regarding methodological limitations due to the reliable ratings across the CASP tool in the main studies informing this finding. Uncertainty as to whether ethical issues were taken into consideration were observed in one study and no ethical issues being taken into consideration were observed in one study. Uncertainty around researcher reflexivity towards their relationship with the research participants was also observed in one of the studies and no consideration for researcher reflexivity in four of the studies. | *No or very minor concerns*  Finding is more descriptive and therefore the primary data is close to that of the finding. There are various issues relating to VR discussed however, such issues all fall under a fear of using the system or issues relating to physical discomfort of the system prior to sustained use. | *Minor concerns*  Minor concerns regarding relevance. A mix of countries, continents, stakeholders and settings. Each study informing this finding met the inclusion criteria of the review and the review finding specified here. Each study adequately referred to caregivers or older adults living with dementias’ concerns regarding fear and physical discomfort of using VR. | *No or very minor concerns*  Each of these studies adequately inform the review finding, all of which report on concerns participants have regarding fears and physical discomfort when using VR. Baker, Hodge, and Rose provide rich accounts supported by observations and quotes from participants. Moyle, Foloppe and Matsangidou provide thin data regarding the preconceptions and causes for refusal in using the VR systems. Although this data is thin in places, the studies as a whole paint a picture of the various ways through which these concerns can arise. Therefore, there is no or very minor concerns about data adequacy for this finding. | **High confidence** | No or very minor concerns regarding methodological limitations, coherence and adequacy, and minor concerns regarding relevance. |
| Finding 2: Education, training and set-up procedures for older adults living with dementia and caregivers can be useful for assessing eligibility, providing reassurance, safety and highlight potential benefits of VR. | [57-60, 63, 68, 71] | *No or very minor concerns*  No or very minor concerns regarding methodological limitations due to the reliable ratings across the CASP tool in the main studies informing this finding. One study was assessed as having serious concerns regarding methodological limitations. However, this study did not contribute to this finding in a way that was considered significant enough to impact the overall assessment of its methodological limitations. | *No or very minor concerns*  The varying ways, and the capacity in which, the caregiver/family member were present during the VR experience were explored in this review finding, with the varying contexts across each of the studies being considered. | *Minor concerns*  Minor concerns regarding relevance. A mix of countries, continents, stakeholders and settings.  Each study informing this finding met the inclusion criteria of the review. | *No or very minor concerns*  Each of these studies adequately inform the review finding, all of which report on some or all of the following: education, training and set-up procedures. Although this data is thin in places, the studies as a whole paint a picture of the various ways through which VR can be facilitated for people with dementia, as well as how their caregivers can help with this. Therefore, there is no or very minor concerns about data adequacy for this finding. | **High confidence** | No or very minor concerns regarding methodological limitations, coherence and adequacy, and minor concerns regarding relevance |
| Finding 3: The continual presence of the caregiver/family member during the VR experience can provide encouragement, motivation and reassurance for older adults living with dementia to use VR. | [58-60, 69-71] | *Moderate concerns*  Mostly reliable ratings across the CASP tool in the studies informing this finding. One study was assessed as having serious concerns regarding methodological limitations due to concerns due to lack of reporting of data collection, analysis, ethical procedures, and reflexivity. | *No or very minor concerns*  The data informing this finding is coherent, as it reports on the different ways family members and caregivers support users when using VR through encouragement, motivation and reassurance. | *No or very minor concerns*  No or very minor concerns regarding relevance. A mix of countries, continents, stakeholders and settings. Although some studies only provided formal and informal caregiver perspectives, these were relevant to the phenomenon of interest and related to the role of the caregiver/family member in order to assist the older adults living with dementia to experience VR. | *Moderate concerns*  Descriptive finding which is supported with limited quantity of data. Most of the studies provided instances where the presence of the caregiver was reported as a motivator and means of reassurance.However, the number of studies and references informing this finding are limited, meaning there are moderate concerns regarding adequacy for this finding. | **Moderate confidence** | No or very minor regarding coherence and relevance, moderate concerns regarding methodological limitations and adequacy. |
| Finding 4: Support and facilitation must be adapted to the needs of older adults living with dementia as they are engaging in VR. This may be through verbal praise and encouragement, physical readjustment or assistance with the system or adapting the virtual environment to suit one’s needs. | [57-60, 63, 64, 69-71] | *Moderate concerns*  Studies vary in terms of their methodological quality; two of the main contributing studies have serious concerns while the remainder have minor and moderate. Moderate concerns regarding methodological limitations were determined due to no or limited ethical issues being taken into consideration in four studies informing this finding. Uncertainty around researcher reflexivity was also observed in one of the studies and no consideration for researcher reflexivity in six of the studies. | *No or very minor concerns*  Physical assistance well described and supported with quotations. Clear indication that physical assistance was required by informal and formal caregivers. Verbal intervention also well described through rich description for some of the included studies. Clear thread between the primary data and that of the finding. | *Minor concerns*  A mix of countries, continents, stakeholders and settings. Formal and informal caregivers’ perceptions and experiences of support and facilitation for older adults living with dementia are provided through interviews and observational data. Limited primary accounts from older adults living with dementia. However, the accounts from formal and informal caregiver are directly related to the phenomenon of interest. | *No or very minor concerns*  Descriptive finding containing large quantity of data. Data provided gives an account of instances of support and facilitation. Based on an overall assessment of the quantity and thinness of the data, we only had minor concerns given the descriptive and broad nature of the finding. | **Moderate confidence** | No or very minor concerns regarding coherence, adequacy, minor concerns regarding relevance, and moderate concerns regarding methodological limitations. |
| Finding 5: Older adults living with dementia can experience a sense of immersion, presence or embodiment in the virtual environment. | [58, 61-63, 66, 71] | *Moderate concerns*  Half of the main contributing reports had moderate concerns regarding lack of reporting on recruitment strategies and ethical issues. The included studies illustrate one serious, one moderate and the remainder are minor overall concerns. | *No or very minor concerns*  The finding is descriptive and primary data is close to the finding. Although extracted data does not use the term immersion, presence, embodiment etc. they relate to the concept of these terms. Although varied experiences of these concepts, they all relate to the overall finding. | *No or very minor concerns*  A mix of countries, continents, stakeholders and settings. Some of the included studies only included a subset of the population when contributing to the review finding. However, their perspective was directly relevant to the research question and the finding. | *Minor concerns*  Descriptive finding with limited scope: Instances where immersion itself is not mentioned but is implied in the accounts of caregivers and older adults living with dementia. Rich description on how immersion, presence and embodiment have been experienced albeit, if participants are unaware of the terminology. Based on an overall assessment of the richness and quantity of the data, we had minor concerns given the descriptive and broad nature of the finding. | **Moderate Confidence** | No or very minor concerns for coherence and relevance, minor concerns regarding adequacy and, moderate concerns regarding methodological limitations. |
| Finding 6: For older adults living with dementia, VR can provide connections to broader experiences beyond where they currently reside. | [58, 60-63, 69, 71] | *Minor concerns*  Main contributing studies illustrate no or very minor concerns. One study was assessed as having serious concerns regarding methodological limitations. Therefore, overall assessment oof minor concerns was considered appropriate. | *No or very minor concerns*  There is a coherent link between the data informing this finding and the finding itself, with the majority of the data referring to how VR facilitated experiences that participants could not experience due to various barriers. | *Minor concerns*  Minor concerns regarding relevance. A mix of countries, continents, stakeholders and settings. Range of stakeholder perspectives which are of direct relevance to the research question. | *No or very minor concerns*  Clear and repetitive pattern of the idea of connection with another outside world. Although this data is thin in places, the studies as a whole paint a picture of the various ways through which VR can help provide connections beyond their physical environment. Therefore, there is no or very minor concerns about data adequacy for this finding. | **High confidence** | No or very minor concerns regarding coherence and adequacy, minor concerns regarding relevance and methodological limitations. |
| Finding 7: VR can provide a means of unlocking memories and connections to the past for older adults living with dementia. | [58-64, 66, 69, 70] | *Moderate concerns*  Two main contributing studies have moderate concerns and one study with serious concerns. Lack of reporting on ethical issues was seen in two studies informing this finding and uncertainty around ethical issues being taken into consideration in one study – with this study being a major contributor to the finding and also being determined to have moderate concerns for methodological limitations. Uncertainty around researcher reflexivity towards their relationship with the research participants was also observed in one of the studies and no consideration for researcher reflexivity in two of the studies. | *No or very minor concerns*  Primary data clear and consistent with the notion of reminiscence and connection to one’s past. Various instances of triggering personal memories explained with reference to specific instances supported by observations and quotations. Data a mostly positive experience but, there is an instance which contradicts this; the finding is broad enough to encompass both without the need to downgrade. | *Minor concerns*  Minor concerns regarding relevance. A mix of countries, continents, stakeholders and settings. Disproportionate number of studies from the UK, accounting for half of the contributing studies. Range of stakeholder views which are of direct relevant to the research question. | *No or very minor concerns*  Consistent data relating to memories and reminiscence. Observations and direct quotations give additional context to data. Based on an overall assessment of limited richness and quantity of the data, we only had no or very minor concerns given the descriptive and broad nature of the finding. | **Moderate confidence** | No or very minor concerns regarding coherence and adequacy, minor concerns regarding relevance and moderate concerns regarding methodological limitations. |
| Finding 8: VR can provide an opportunity for shared experiences and can enhance the social connection and engagement of older adults living with dementia. | [58, 60-64, 66, 67, 69, 71] | *Moderate concerns*  Moderate concerns regarding methodological limitations were determined due to no ethical issues taken into consideration in three studies, one of which was a major contributor to the finding. Uncertainty around researcher reflexivity towards their relationship with the research participants was also observed in eight of the studies*.* | *No or very minor concerns*  Clear instances of social aspects demonstrated through increased verbalizations and opportunities for conversation based on VR reflections and anticipation for future use. | *Minor concerns*  Minor concerns regarding relevance. A mix of countries, continents, stakeholders and settings. Studies meet the inclusion criteria and provide a multi-stakeholder perspective. Although, there are instance where primary data from older adults living with dementia are not provided, data from other stakeholders is of direct relevance to the findings and overall research question. | *No or very minor concerns*  Clear repetition of the concept of increased social interaction and engagement. Supported by observations and quotes from participants. One study provided thin data. Although this data is thin in places, the studies as a whole paints a picture of the various difference ways through which VR can help provide connections with other people. Therefore, there is no or very minor concerns about data adequacy for this finding. | **Moderate confidence** | No or very minor concerns regarding coherence and adequacy, minor concerns regarding relevance and moderate concerns regarding methodological limitations. |
| Finding 9: When the level of interaction is suited to the abilities of older adults living with dementia they may achieve a sense of agency, empowerment and control in the VE. However, when the level of interaction is not suited the opposite may be experienced. | [58-60, 62, 63, 66, 69, 71] | *Moderate concerns*  Moderate concerns regarding methodological limitations were determined due to no ethical issues taken into consideration in two studies and uncertainty around ethical issues being taken into consideration in one study, which provided a moderate contribution to the finding. No consideration for researcher reflexivity was found in six of the studies. | *Minor concerns*  Control, freedom and empowerment directly mentioned in the primary data; may be some ambiguity between the association of control to level of interaction. Contradictory primary data is explored in the finding and therefore, there is no need to downgrade the one instance of disempowerment. | *No or very minor concerns*  No or very minor concerns regarding relevance. A mix of countries, continents, stakeholders and settings. Studies relevant to the finding and the overall research question. Studies meet the eligibility criteria. | *Minor concerns*  Each of these studies adequately inform the review finding, all of which report on some or all of the following: a sense of agency, empowerment and control in the VE. However, the number of studies and references informing this finding are limited, meaning there are minor concerns regarding adequacy for this finding. | **Moderate confidence** | No or very minor concerns regarding relevance, minor concerns regarding adequacy and coherence and moderate concerns regarding methodological limitations. |
| Finding 10: Older adults living with dementia may demonstrate dynamic use of VR. This includes varying length of use, levels of interaction, difficulty and tolerability of the system. | [57-71] | *Moderate concerns*  Moderate concerns regarding methodological limitations were determined due to no ethical issues taken into consideration in four studies – one of which provided a moderate contribution to the finding – and uncertainty around ethical issues being taken into consideration in two studies. No consideration for researcher reflexivity observed in 10 of the studies. | *No or very minor concerns*  Descriptive finding  The variance of data can be categorized into difficulty using the system (body tracking, controllers, HMD etc.), length and level of VR use. Clear reference of instances these when engaging with VR. Inclusion of observations and quotations to support findings. | *No or very minor concern*  No or very minor concerns regarding relevance. A mix of countries, continents, stakeholders, and settings. Range of stakeholder views. Although, not always primary data from the older adult living with dementia, other stakeholder perspectives are of direct relevance to the research question. | *No or very minor concerns*  Given the descriptive and broad nature there is an adequate quantity of data, despite limited richness of some extracts. Observations and quotations provided also.  Based on an overall assessment of the richness and quantity of the data, we only had no or very minor concerns given the descriptive and broad nature of the finding | **Moderate confidence** | No or very minor concerns regarding coherence, relevance, adequacy and, moderate concerns regarding methodological limitations. |
| Finding 11: VR can provide an ‘enlivening’ experience for older adults living with dementia. Enjoyment, happiness, laughter, awe, positive mood, sensory stimulation, excitement and surprise may be exhibited when using VR. | [57-64, 66, 67, 69-71] | *Moderate concerns*  Minor concerns regarding methodological limitations were determined due to the mostly reliable ratings across the CASP tool in the studies informing this finding. Two studies with contributions to this finding were determined to have moderate and serious concerns regarding methodological limitations, respectively. | *No or very minor concerns*  Varied primary data presented. However, due to the broad nature of the finding, each instance falls under one of the subgroups presented. Observations and quotations presented dissipate ambiguity surrounding the enlivening response of the system. | *No or very minor concerns*  No or very minor concerns regarding relevance. A mix of countries, continents, stakeholders and settings. Although, not always primary data from the older adult living with dementia, other stakeholder perspectives are of direct relevance to the research question. | *No or very minor concerns*  Consistency across the finding of the idea of an enlivening experience. Such an experience is accounted for by the subgroups identified in the finding. Three studies provide thin data. Based on an overall assessment of the limited thickness and quantity of the data, we had no or very minor concerns given the descriptive and broad nature of the finding. | **Moderate confidence** | No or very minor concerns regarding coherence, relevance, adequacy and, moderate concerns regarding methodological limitations. |
| Finding 12:  VR may provide a soothing and calming experience for some older adults living with dementia. | [59-62, 66, 67, 71] | *Minor concerns*  Minor concerns regarding methodological limitations were determined due to the mostly reliable ratings across the CASP tool in the studies informing this finding. Two studies with minor contributions to this finding were determined to have moderate and serious concerns regarding methodological limitations. | *No or very minor concerns*  Descriptive finding with  varied primary data presented. Clear instances of VR providing a calming experience through visual and auditory stimulation. Quotations and observations provide a clear link to the finding and remove ambiguity. | *No or very minor concerns*  No or very minor concerns regarding relevance. A mix of countries, continents, stakeholders and settings. Studies meet the inclusion criteria and are of direct relevance to the finding and the research question. | *Minor concerns*  Each of these studies adequately inform the review finding, all of which report on some or all of the following: VR can provide a soothing and calming experience for some people living with dementia. However, the number of studies and references informing this finding are limited, meaning there are minor concerns regarding adequacy for this finding. | **High confidence** | No or very minor concerns regarding coherence and relevance, minor concerns regarding adequacy and methodological limitations. |
| Finding 13: VR use may result in negative emotions and sensations for older adults living with dementia including: dizziness, disorientation, BPSD symptoms and fatigue. | [57-59, 61-63, 65-68, 70, 71] | *Serious concerns*  Serious concerns regarding methodological limitations for main contributor of this finding. There were no ethical issues taken into consideration in three studies and uncertainty around ethical issues being taken into consideration in two studies. No consideration for researcher reflexivity found in ten of the studies. | *No or very minor concerns*  The range of negative experiences reported in this finding is coherent with the data that supports it. | *No or very minor concerns*  No or very minor concerns regarding relevance. A mix of countries, continents, stakeholders and settings.  Range of stakeholder perspectives. Although, not always primary data from the older adult living with dementia, other stakeholder perspectives meet inclusion criteria and are of direct relevance to the research question. | *No or very minor concerns*  Each of these studies adequately inform the review finding, all of which report on some or all of the following: negative emotions and sensations for people living with dementia. Although this data is thin in places, the studies as a whole paint a picture of the various ways through which people with dementia experienced negative emotions and sensations. Therefore, there is no or very minor concerns about data adequacy for this finding. | **Moderate confidence** | No or very minor concerns regarding coherence, relevance and adequacy, and serious concerns regarding methodological limitations. |
| Finding 14: VR effects may be translated into the daily lives and routine of older adults living with dementia. Improvements in cognition, memory, concentration sustained attention, improved task organisation, motivation, positive mood and overall wellbeing may be perceived after VR sessions. | [59, 61-63, 65, 66, 68, 69] | *Minor concerns*  Minor concerns regarding methodological limitations were determined due to the mostly reliable ratings across the CASP tool in the studies informing this finding. One study with moderate contributions to this finding was determined to have moderate concerns regarding methodological limitations. | *No or very minor concerns*  Varied primary data presented. However, due to the broad nature of the finding, each instance falls under one of the subgroups presented. | *No or very minor concerns*  No or very minor concerns regarding relevance. A mix of countries, continents, stakeholders and settings. Range of stakeholder perspectives. Although, not always primary data from the older adult living with dementia, other stakeholder perspectives meet inclusion criteria and are of direct relevance to the research question. | *Minor concerns*  Each of these studies adequately inform the review finding, all of which report on VR effects which can be translated into the daily lives and routine of older adults living with dementia. However, the number of studies and references informing this finding are limited, meaning there are minor concerns regarding adequacy for this finding. | **High confidence** | No or very minor concerns regarding coherence and relevance, minor concerns regarding adequacy and methodological limitations. |
| Finding 15: Older adults living with dementia can exhibit a spectrum of willingness to try VR again indicated by positive, neutral, and negative comments. Older adults with dementia can share their positive experiences and anticipation with their peers and community. | [57-61, 64-67, 69, 71] | *Moderate concerns*  Moderate concerns regarding methodological limitations were determined due to serious concerns regarding methodological limitations for the main contributor to this finding. There were no ethical issues taken into consideration in three studies. No consideration for researcher reflexivity observed in nine of the studies. | *Minor concerns*  There is coherence between the finding and the data supporting it with regards to the spectrum of willingness to try VR again. The varying reasons for this willingness or unwillingness were explored in this finding. However, some instances may not directly relate to a willingness to try VR again. | *No or very minor concerns*  No or very minor concerns regarding relevance. A mix of countries, continents, stakeholders and settings. All studies satisfy the inclusion criteria and research question. | *No or very minor concerns*  Each of these studies adequately inform the review finding, all of which report on exhibiting a spectrum of willingness to try VR again. Although this data is thin in places, the studies as a whole paint a picture of the various ways through which people with dementia expressed their willingness or unwillingness to try VR again. Therefore, there is no or very minor concerns about data adequacy for this finding. | **Moderate confidence** | No or very minor concerns regarding relevance and adequacy, minor concerns regarding coherence, and moderate concerns regarding methodological limitations. |
| Finding 16: Formal and informal caregivers may identify a change in their attitude toward VR and show new learning of the capabilities of the older adults living with dementia after observing older adults living with dementia use the system. | [59-63, 69, 71] | *Minor concerns*  Minor concerns regarding methodological limitations were determined due to the mostly reliable ratings across the CASP tool in the studies informing this finding. Two studies with minor contributions to this finding were determined to have moderate and serious concerns regarding methodological limitations, respectively. | *No or very minor concerns*  There is coherence between the finding and the data supporting it with regards to caregivers’ attitudes towards VR with the data representing what is reported in the finding. | *Minor concerns*  Minor concerns regarding relevance. A mix of countries, continents, stakeholders, and settings.  Disproportionate number of studies from the UK. Although, not always primary data from the older adult living with dementia, other stakeholder perspectives meet inclusion criteria and are of direct relevance to the research question. | *Minor concerns*  Each of these studies adequately inform the review finding, all of which report on some or all of the following: a change in their attitude toward VR and show new learning of the capabilities of the older adults with dementia. However, the number of studies and references informing this finding are limited, meaning there are minor concerns regarding adequacy for this finding. | **High confidence** | No or very minor concerns regarding coherence, minor concerns regarding methodological limitations, relevance, and adequacy. |
