# Supplementary material for: Key Stakeholders’ Experiences and Perceptions of Virtual Reality for Older Adults Living With Dementia: Systematic Review and Thematic Synthesis
Source: JMIR Serious Games. 2022 Dec 23;10(4):e37228. doi: 10.2196/37228 (PMC9823606; doi:10.2196/37228)
Supplement: Multimedia Appendix 8 [file games_v10i4e37228_app8.docx]

**Multimedia Appendix 7: Supporting Quotations**

| Analytical themes | Subtheme | Quotation |
| --- | --- | --- |
|  |  |  |
| **Stepping into virtuality** |  |  |
|  | Stepping into the unknown | “I can't imagine that she has the patience to hold something like (Google Cardboard) that for a long time” [60]  “Caregivers were unsure whether people with dementia would try HMD-VR at all.” [61]  “Because of the material factors of the system, three out of ten PwD […] refused to put on the HMD.” [70] |
|  | Supporting the step into virtuality | “We were given a troubleshooting guide. It went through the step by step how to get it started and then it’s also got a section in there for if something’s not working” [59]  “we brought along a set of pictures to display the VR experience in its entirety before asking participants if they would like to try”… “Part of this process was also demonstrating the different ways of using the VR system-through a head-mounted display, and through a handheld Google Cardboard display.” [60]  “With encouragement from her daughter it seemed a lot easier to ask Lucy to try it [HMD] on” [60]  “We really had to take over the role of the motivator.” [69]  “I think by having a nurse and a physiotherapist come in, it gives a bit of reassurance for the resident” [58] |
| **Escape to virtuality** |  |  |
|  | An immersive world | “I can already imagine that I’m with the cows, rabbits, giving them food, playing with them behind the display.” [66]  “ They reported that HMD-VR felt ‘real’ and they felt like they were ‘in’ the VE (Extract 15). […] I felt like I was in the beach… it was very good feeling. (Person with dementia 4, 7, 141-144)” [61]  “VWs not only allowed the resident to remember particular objects (flowers, trees, buildings etc.), but also triggered the overall feeling of “being in a garden.” [63]  “Most participants reported a high level of presence during the interviews, reporting that it felt “real” or “like they were in there”” [62] |
|  | Unlocking and Maintaining Connections | “Michael and Linda indicated that a VR system could act as a missing link between their current situation –having to depend on public transport –and their past, where they visited many favourite places together.” [60]  “Person with dementia thought HMD-VR would be a good way to see what going abroad might be like, ‘because if you’re going abroad, all you get is a video of what’s going to be like’” [61]  “We believe that virtual worlds provide a means for them [older adults living with dementia] to reconnect their experience to the “day-to-day” yet “extraordinary” worlds.” [63]  ““I am in Disneyland again! I am close to the ice-mountains!” Researcher intervene: “Have you been at Disneyland?” [PwD giggles and nods her head:] “Long time ago when my children were still young!”[Patient 7, HCI Researcher] ” [70]  “VWs [virtual worlds] allow the residents to temporarily step outside of their closed physical environment of long-term care facilities and transport them to a (albeit virtual) world of reminiscence” [63]  “The interaction… because we have got two levels, those downstairs never meet people from upstairs… and they got to know each other ” [58]    “Many of the participants reported that the afternoon tea sessions at Bluebell Grove were an opportunity to spend meaningful time in public with their loved ones, but also to access social support and advice from professional carers and from others facing the challenges of dementia.” [60] |
|  | Interaction and Empowerment | “the installation gives them a feeling of being in the control and meaningfulness.” [66]  “Running counter to initial reticence around trying out the VR environments is a sense of freedom and enjoyment seemingly enjoyed by participants once they tried the headset and the environment[…]This freeing effect is clearly pleasurable for participants who can engage in enjoyable activities” [60]  “One participant was very clear in what they did not like (“I’ve got no real control. There’s no music. I can’t make it tilt (Sic)” [RM1]).” [59]  “Mum was using her hands to control the movement. It means she’s got control of something in her life, that control element. What other control has she really got?” [FF6]…“Their faces light up when they also realise that they’re controlling what’s happening on the screen” [SF2] [59] |
|  | Physical, Cognitive, Affective | “They noticed the elevated mood of some residents and expressed the positive influence it has in their everyday life” [66]  “Straight away [Janet] started to sing. It sounded to me that she was trying to repeat the lyrics ... ” [60]  “The majority of the residents seem to really enjoy it. I see their faces smiling, and they seem quite relaxed with it” [SF1] [59]  “People with dementia reported that they found HMD-VR ‘fun’ and ‘quite exciting…you never know what’s beyond the corner, do you?’ (Extract 4, Person with dementia 13, 4, 79-80).”[61]  “We were informed this PWD could become agitated easily, and yet surprisingly, he tolerated VR and used it for the maximum period.”[PWD7, Observations, 12] [62]  “Post VR Observations [..]Commented, "It was the best day ever". Talked to others including peers and CGs commenting, "Best day I've ever had."[PWD3, Observations, 1] [62] |
| **Returning to reality:** **Reflecting on the virtual experience** |  | ““Nice of you to do this for me” “this is so nice”[..]“It was alright; wasn’t good and wasn’t bad”—Do again: “over and over” […] “Do Again: “Depends on the program” “It’s a one-time experience, you don’t need it twice”; “What else do I get to see?” – Said they just didn’t enjoy it – “Just not that interesting to me. Sorry I couldn’t help you” – “Interesting ... nothing I can’t live without.” [67]  “Although Ruby mentioned that she could see others using the system, she stated a preference for the “real thing.””[60]  “When asked if she would continue to use the system after the study ended, she replied “yes, it is a good memory training and it gives it a structure to everyday life.” [69] |
